# Monkey multi-organ cell atlas exposed to estrogen

**DOI:** 10.1093/lifemedi/lnae012

**Published:** 2024-03-22

**Authors:** Wen Fang, Jiao Qu, Wanjun Zhao, Xinran Cao, Jinran Liu, Quan Han, Dijun Chen, Wen Lv, Yicheng Xie, Yang Sun

**Affiliations:** State Key Laboratory of Pharmaceutical Biotechnology, School of Life Sciences, Nanjing University, Nanjing 210023, China; State Key Laboratory of Pharmaceutical Biotechnology, School of Life Sciences, Nanjing University, Nanjing 210023, China; Jiangsu Key Laboratory of New Drug Research and Clinical Pharmacy, Xuzhou Medical University, Xuzhou 221004, China; State Key Laboratory of Pharmaceutical Biotechnology, School of Life Sciences, Nanjing University, Nanjing 210023, China; State Key Laboratory of Pharmaceutical Biotechnology, School of Life Sciences, Nanjing University, Nanjing 210023, China; State Key Laboratory of Pharmaceutical Biotechnology, School of Life Sciences, Nanjing University, Nanjing 210023, China; State Key Laboratory of Pharmaceutical Biotechnology, School of Life Sciences, Nanjing University, Nanjing 210023, China; State Key Laboratory of Pharmaceutical Biotechnology, School of Life Sciences, Nanjing University, Nanjing 210023, China; Department of Gynecology, Tongde Hospital of Zhejiang Province, Hangzhou 310012, China; Children’s Hospital, Zhejiang University School of Medicine, National Clinical Research Center for Child Health, Hangzhou 310052, China; State Key Laboratory of Pharmaceutical Biotechnology, School of Life Sciences, Nanjing University, Nanjing 210023, China; Jiangsu Key Laboratory of New Drug Research and Clinical Pharmacy, Xuzhou Medical University, Xuzhou 221004, China

**Keywords:** estrogen, single-cell RNA sequencing, single-cell ATAC sequencing, cynomolgus monkeys

## Abstract

Awareness of estrogen’s effects on health is broadening rapidly. The effects of long-term high levels of estrogen on the body involve multiple organs. Here, we used both single-cell chromatin accessibility and RNA sequencing data to analyze the potential effect of estrogen on major organs. The integrated cell map enabled in-depth dissection and comparison of molecular dynamics, cell-type compositions, and cellular heterogeneity across multiple tissues and organs under estrogen stimulation. We also inferred pseudotime cell trajectories and cell–cell communications to uncover key molecular signatures underlying their cellular processes in major organs in response to estrogen. For example, estrogen could induce the differentiation of *IFIT3*^*+*^ neutrophils into *S100A9*^*+*^ neutrophils involved in the function of endosome-to-lysosome transport and the multivesicular body sorting pathway in liver tissues. Furthermore, through integration with human genome-wide association study data, we further identified a subset of risk genes during disease development that were induced by estrogen, such as *AKT1* (related to endometrial cancer), *CCND1* (related to breast cancer), *HSPH1* (related to colorectal cancer), and COVID-19 and asthma-related risk genes. Our work uncovers the impact of estrogen on the major organs, constitutes a useful resource, and reveals the contribution and mechanism of estrogen to related diseases.

## Introduction

Estrogens are among the most ubiquitous and important hormones in the female body [1, 2]. Estrogen can affect female reproductive function [3, 4]. However, the biological response produced by estrogens is not limited to effects on reproduction [5–7]. Estrogen travels far, interacts with multiple organ systems, and plays a pivotal role in sentinel physiologic events [8–10].

In recent years, the effects of high levels of estrogen on the body have gradually attracted the attention of scientists. Prolonged high levels of estrogen in the body can lead to the progression of related diseases such as endometrial hyperplasia [11]. Estrogen exerts its biological function mainly through estrogen receptors (ERs) [12, 13]. ERs are widely distributed in organs such as the uterus, lung, liver, colon, and heart [14–18]. This suggests, to some extent, that estrogen may have a potential effect on all organs that have estrogen receptor distribution. Many studies have also confirmed that high levels of estrogen can affect the progression of many diseases [19]. However, there is relatively little research on how estrogen affects multiple organ systems. This may be due to the fact that there is no way to collect suitable tissues from the clinic that are exposed to high levels of estrogen. Non-human primates are phylogenetically close to humans [20, 21]. Therefore, it is of great significance to use cynomolgus monkeys to explore the effects of high levels of estrogen on major organs. Rapid advances in single-cell multi-omics technologies have enabled molecular quantification of thousands of cells at once, leading to meticulous insight into organ compositions and molecular mechanisms driving cellular heterogeneity [22–24]. This will help us provide more information about the effects of estrogen on multiple tissues at single-cell levels.

Thus, we used single-cell multi-omics technologies to explore the dissection and comparison of molecular dynamics, cell-type compositions and cellular heterogeneity across multiple tissues and organs in response to estrogen. We also analyzed human genome-wide association studies (GWASs) and identified high-risk disease genes that become dysregulated in organs in response to estrogen. This study provides an extensive resource on estrogen-driven changes in major organs and identifies genes that potentiate the risk of estrogen-associated disorders.

## Results

### Multi-organ cell atlas by multi-omic analysis in estrogen-triggered monkey

**Figure 1.**
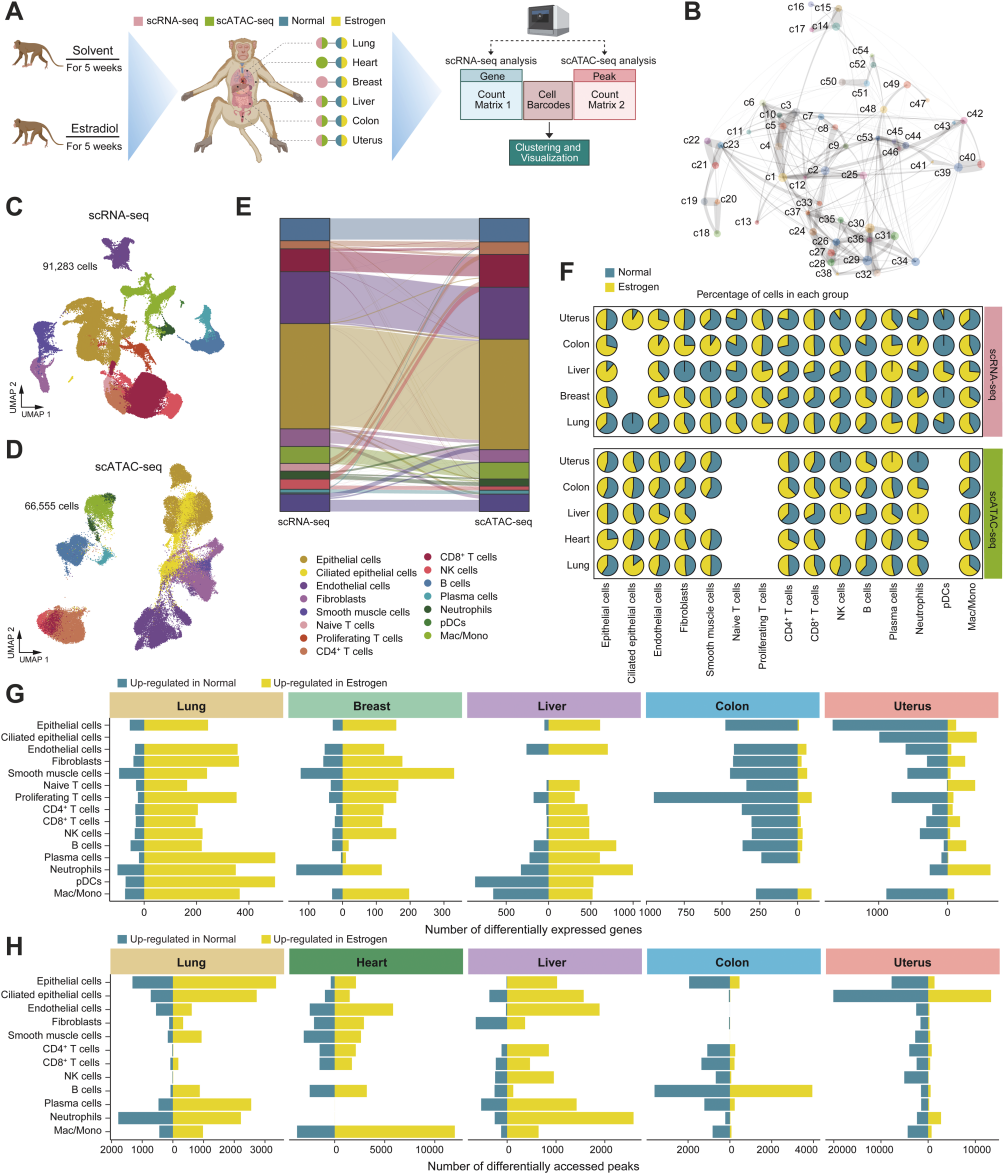
**Multi-organ single-cell landscape of cynomolgus monkeys in normal and estrogen groups.**(A) Workflow of sample collection and data analysis in this study. (B) Constellation plot depicting the global relatedness among all sub-clusters. Each cluster is represented by a dot, positioned at its centroid. (C) UMAP plot displaying the integrated cell map based on scRNA-seq data, consisting of 91,283 cells from 15 annotated cell types. Cells are colored by cell types. (D) UMAP plot displaying the integrated cell map based on scATAC-seq data, which consists of 66,555 cells from 12 annotated cell types. Cells are colored by cell types. (E) Sankey diagram showing the consistency of cell type annotations between scRNA-seq and scATAC-seq data. (F) Scatter pie plot illustrating the proportion of normal and estrogen groups within each cell type of each organ. (G) Bar plot showing the number of differentially expressed genes (DEGs) between the normal and estrogen groups within each cell type of each organ. The calculation process for DEGs is akin to first extracting each organ from the integrated samples, then extracting each cell type from each organ, and finally calculating the DEGs between groups for each cell type of each organ (FDR < 0.05 and log_2_FC > 0.25). (H) Bar plot showing the number of differentially accessed peaks between the normal and estrogen groups within each cell type of each organ. Detailed computational methods and filtering thresholds for the differentially accessed peaks are as (G).

To determine how estrogen affects organs, we performed single-cell RNA sequencing (scRNA-seq) and single-cell ATAC sequencing (scATAC-seq) in multiple tissues from cynomolgus monkeys with or without estrogen treatment (Figs. 1A and S1). We correct for batch effects by integrating all scRNA-seq data using robust principal component analysis (RPCA). After removing low-quality cells, we obtained 91,283 cells for subsequent analysis (Fig. S2). We explored the relationships among the 54 clusters by visualization with Uniform Manifold Approximation and Projection (UMAP) and constellation plots (Fig. 1B). And unsupervised clustering using UMAP resolved major cell types based on well-established markers, including unciliated epithelial cells, ciliated epithelial cells, endothelial cells, fibroblasts, smooth muscle cells, and immune cells (Figs. 1C and S3).

The scATAC-seq data also comprised paired samples from the normal and estrogen-treated groups across five organs. Based on enrichment analysis of accessible DNA sequences relative to the TSS and unique fragment size distribution, scATAC-seq data were high-quality (Fig. S4). After batch effect correction with Harmony in ArchR, UMAP clustering analysis of the integrated scATAC-seq data revealed major cell types (Fig. 1D), which were annotated based on chromatin accessibility at well-characterized marker gene promoter regions. The consistency of scATAC-seq and scRNA-seq data for cell type annotation was also explored. We first performed cross-modality integration analysis in organ-matched samples between RNA and ATAC. We then assigned the cell type of each cluster using labels annotated from scRNA-seq data and identified major cell types in the RNA-ATAC integration (Fig. S5). Major cell types identified by scRNA-seq and scATAC-seq data are highly consistent (Fig. 1E). These results highlight the quality of the dataset. Then we compared normal and estrogen groups in different cell types and organs using scRNA-seq and scATAC-seq data (Fig. 1F). Subsequently, we analyzed the differential genes in different cell types from different organs between the two groups. Estrogen affects the differential gene in almost all cell types of the lung, breast, and liver. The estrogen group had more upregulated genes and peaks compared to the normal group, whereas the colon and uterus showed the opposite trend (Fig. 1G and 1H).

### Common mechanisms of estrogen response in different organs

**Figure 2.**
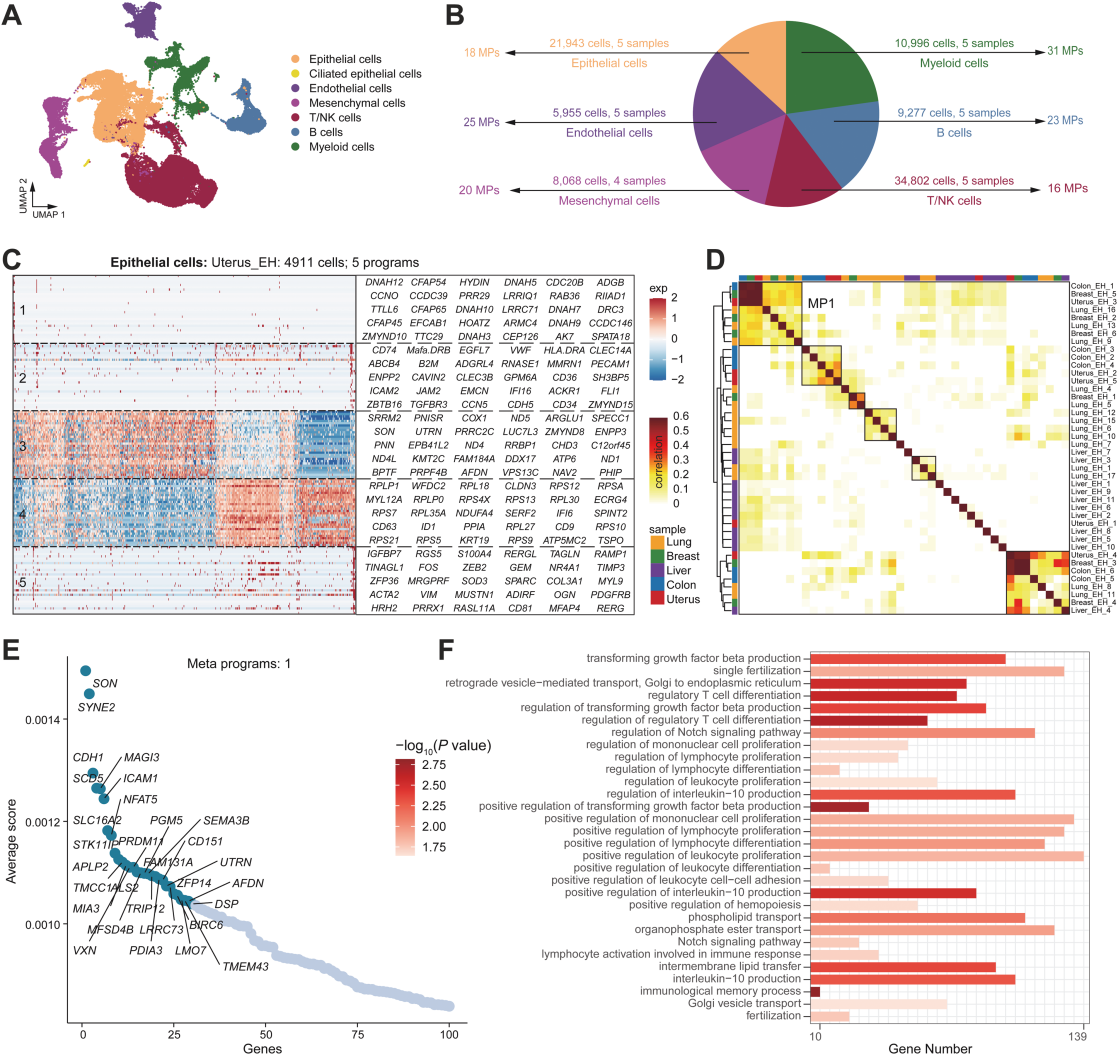
**Unbiased clustering reveals MPs that collectively respond to the estrogen signaling within each organ across various major cell types.**(A) UMAP showing the distribution of major cell types. Cells are colored by major cell types. (B) Pie chart depicting the annotation of all major cell types in the compendium. Each color corresponds to a major cell type, and an arrow indicates the corresponding number of cells, samples, and MPs. (C) Heatmap showing gene expression programs deciphered from epithelial cells of a representative sample (Uterus_EH) using non-NMF. (D) Heatmap showing Pearson correlation indices for comparisons among 42 NMF programs based on their top 50 genes. Programs are ordered by clustering and grouped into MPs (marked by black solid lines). (E) Scatter plot showing the top 100 genes of MP1 ordered by average specific score, and the top 30 genes with high scores are highlighted. (F) Bar plot displaying the functional pathways enriched by the top 100 genes in (E).

We next explored how expression states varied across organs within the same cell type (Fig. 2A). We defined these meta-programs (MPs) within these cell types using non-NMF, revealing coherent sets of genes preferentially co-expressed by subsets of cells from different organs (Fig. 2B). First, we filtered five gene signatures that vary among Uterus_EH epithelial cells (Fig. 2C). The approach for each of the five organ samples yielded 42 coherently varying programs across cells in at least one organ. Next, we used hierarchical clustering to distill these 42 programs into meta-signatures that reflect common expression programs that vary within different organs (Fig. 2D). MP1 was particularly emphasized because of its high consistency across organs, indicating a shared pattern of responses to estrogen stimulation. Subsequently, we computed the genes specifically expressed in MP1 (details on the calculation method can be found in the methods section), extracted the top 100 genes, and highlighted the top 30 (Fig. 2E). After Gene Ontology (GO) functional enrichment analysis for the top 100 genes, we presented the top 30 enriched functional pathways (Fig. 2F). We found that MP1 is primarily associated with cell proliferation and differentiation, with some features related to inflammatory responses. Similarly, we identified 37 programs in mesenchymal cells, grouped into 20 MPs. MP1 was linked to cell adhesion, cytoskeleton organization, angiogenesis, muscle development, and tissue morphogenesis (Fig. S6A–D). In endothelial cells, 43 programs were categorized into 25 MPs. Specifically, MP2 was associated with angiogenesis, blood vessel development, cell differentiation, cell signaling, and tissue development (Fig. S6E–H). T/NK cells had 43 defined programs in 16 MPs, with MP1 primarily linked to T cell proliferation and activation (Fig. S7A–D). B cells exhibited 49 programs in 23 MPs. Estrogen induced changes in endoplasmic reticulum stress-related pathways in B cells (Fig. S7E–H), potentially impacting pathogen resistance. Myeloid cells presented 67 programs in 31 MPs (Fig. S8). MP1 was tied to neutrophil migration and chemotaxis, MP2 benefited apoptosis and cell death, and MP4 helped regulate cytokine production and myeloid leukocyte migration.

### GRNs for estrogen response in different organs

**Figure 3.**
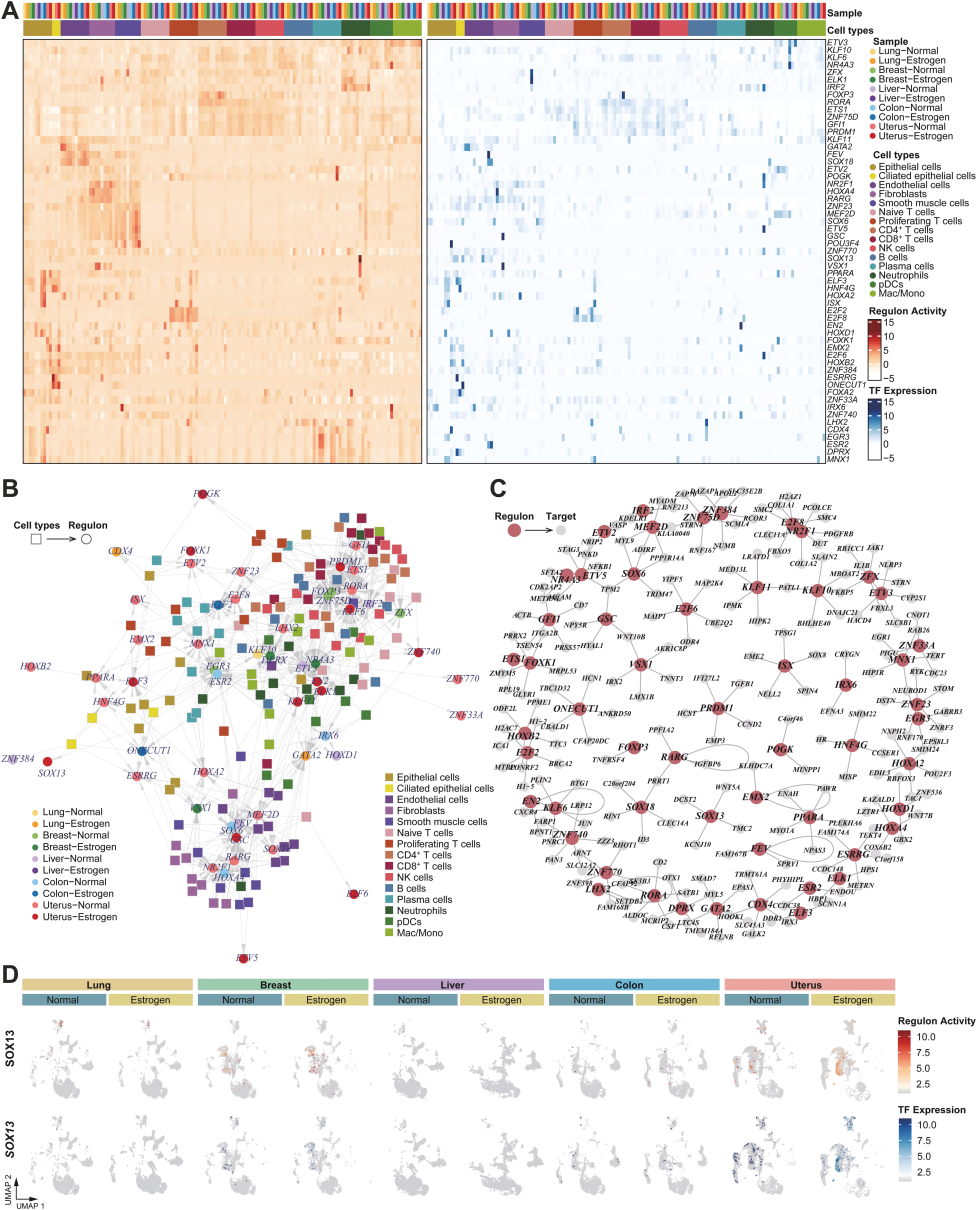
**GRNs for estrogen response in different organs.**(A) Heatmaps showing the regulon activity per cell type in each sample (left) and the expression specificity of the corresponding regulon TFs (right). (B) Network showing the top five regulons for a specific cell type. Regulon-associated cell types are highlighted in squares and regulons in circles. Regulons (in circles) are colored according to their highly expressed samples. (C) A network view of the top five regulons and the top five target genes per regulon. Small nodes denote target genes, and large nodes denote regulons. (D) UMAP depicts the activity and expression specificity of SOX13 in different samples.

Next, we explored specific gene regulatory networks and the activities of transcription factor (TF) combinations in response to estrogen in different organs. We identified TF regulons based on co-expression and motif enrichment, observing notable conformance of these regulons within the same cell types across different organs (Figs. 3A and S9). This suggests a potential systemic regulatory mechanism of estrogen across various organs. In the organ-specific gene regulatory networks, particularly interesting was the discovery that regulons of ETV2 and POGK were specifically upregulated in the uterus of the estrogen group. This finding is of significantly interest, considering the association of ETV2 and POGK with tumor angiogenesis and poor prognosis [25, 26], suggesting a possible link between elevated estrogen levels and an increased risk of endometrial cancer. This highlights the critical need to understand estrogen’s role in uterine health and its implications for cancer development. Furthermore, in the cell-type-specific gene regulatory networks, we found that SOX13 was a specific regulon in uterine epithelial cells in the estrogen group (Fig. 3B). According to a previous study, SOX13 could promote cancer cell proliferation, migration, and chemoresistance [27, 28]. SOX13 was shown to regulate *WNT5A*, *DCST2*, *KCNJ10*, and *TMC2* under estrogen stimulation (Fig. 3C). As reported in earlier research, *WNT5A* could be considered as a potential marker of molecular changes that take place during endometrial cancer development [29–31]. This offers a more comprehensive view of the estrogen-induced regulatory landscape in uterine epithelial cells. Finally, we validated the organ-specific TF regulons using organ-matched scRNA-seq data. The results confirmed that the SOX13 regulon was uterus-specific, with TF deviation scores of SOX13 exhibiting relatively high levels following estrogen treatment in the uterus (Fig. 3D). This further reinforces the notion of organ-specific responses to estrogen, emphasizing the importance of understanding these responses in the context of organ-specific health and disease.

### Disease-associated genes are induced by estrogen

**Figure 4.**
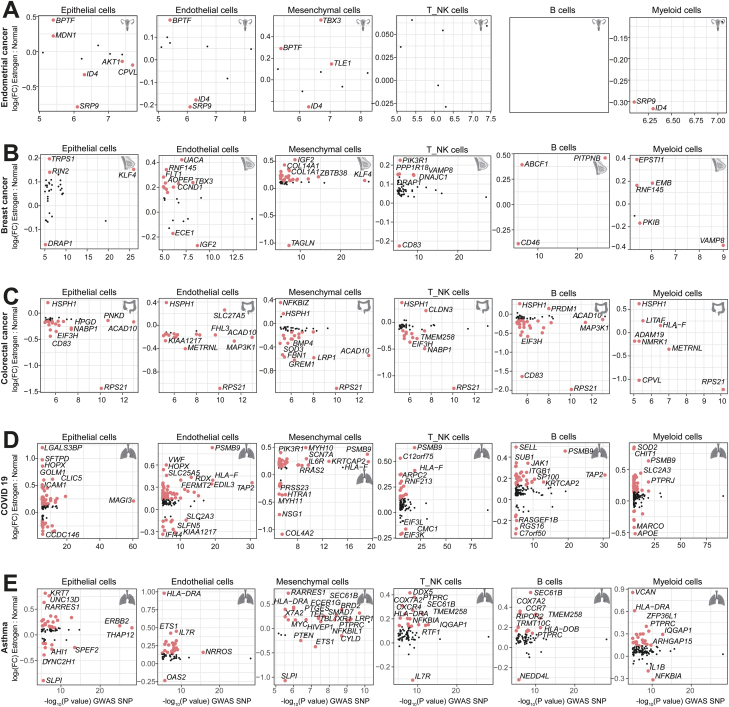
**Integration of human GWAS data reveals risk genes for various diseases induced by estrogen.**(A) Comparing known high-risk variants for endometrial cancer (identified via GWAS) with induced gene expression changes in various cell types in the uterus after estrogen treatment. (B) Comparing known high-risk variants for breast cancer (identified via GWAS) with induced gene expression changes in various cell types in the breast after estrogen treatment. (C) Comparing known high-risk variants for colorectal cancer (identified via GWAS) with induced gene expression changes in various cell types in the colon after estrogen treatment. (D) Comparing known high-risk variants for COVID-19 (identified via GWAS) with induced gene expression changes in various cell types in the lung after estrogen treatment. (E) Comparing known high-risk variants for Asthma (identified via GWAS) with induced gene expression changes in various cell types in the lung after estrogen treatment.

To identify estrogen-induced differentially expressed genes (DEGs) which can increase the risk of organ dysfunction, we integrated our datasets with the NHGRI-EBI GWAS database. We focused on genetic risk loci associated with estrogen-related diseases. Gene loci associated with endometrial cancer, including *BPTF* and *MDN1*, were upregulated in uterine epithelial cells after estrogen treatment (Fig. 4A). Interestingly, the bromodomain PHD finger TF, *BPTF*, is the largest member of the human nucleosome remodeling factor (NURF). Many studies have already discovered that the *BPTF* proliferates in multiple tumors and is increasingly being identified as a protumorigenic factor [32–34]. These observations strongly suggest that estrogen may induce genetic variants of *BPTF*, thereby increasing the risk of endometrial cancer. However, few endometrial cancer-related genes have been identified in immune cells. This suggests that estrogen mainly affects the expression of related genes in uterine epithelial cells and promotes the disease process. Similar phenomena have been demonstrated in other gynecological diseases. *CCND1*-driven and *IGF2*-driven breast cancer have been widely reported [35, 36]. Estrogen led to the upregulation of *CCND1* and *IGF2* in endothelial cells and mesenchymal cells in breast cells from the estrogen group (Fig. 4B). In addition, estrogen-induced increased expression of genes related to collagen synthesis, including *COL14A1* and *COL1A1* in mesenchymal cells, which was associated with cancer cell growth, invasion, metastasis, and angiogenesis [37]. In colon tissue, we noted regulatory effects of estrogen on colon cancer-related risk genes such as *RPS21* and *HSPH1*. Estrogen suppressed *RPS21* expression but increased *HSP11* expression in almost all cell types (Fig. 4C). Both *RPS21* and *HSPH1* have been reported to be involved in tumor progression [38, 39]. While evidence of *HSPH1* associating with colon cancer abounds, current research on *RPS21* in colon cancer, regarding its expression levels, functions, and potential therapeutic targets, is not yet comprehensive. This implies a complex role for estrogen in the colon. Interestingly, we also noticed that estrogen-induced the expression of many of the COVID-19 and asthma-related risk genes involving *HLA-DRA*, *HLA-F*, and *HLA-DOB* (Fig. 4D and 4E), which play an important role in the immune response [40, 41].

### Estrogen promotes the interaction between immune cells in lung tissue

The lung is an important organ due to the fact that it interfaces with the environment across a continuous epithelium composed of various cell types along the proximal and distal airways [42]. We identified 22,261 cells and 15 major clusters from the lung tissues by scRNA-seq (Fig. S10A) and noticed a much greater frequency of intercellular connections between immune cells in estrogen group (Fig. S10B). Next, we identified DEGs that were increased in estrogen group including *S100A8*, *S100A9*, *IFI6*, *IFI27*, *GZMB*, and *SOD2*. (Fig. S10C). Most of these genes were expressed in immune cells (Fig. S10D), especially in Mac/Mono and neutrophils (Fig. S10E). Studies showed that they were almost inflammation-related genes, involved in a variety of biological functions such as immunoregulation of the body [43–45]. This suggests that estrogen may mainly affect the function of immune cells. GSEA revealed that DEGs of estrogen group involved in immune and inflammatory responses (Fig. S10F and S10G). To chart the rewiring of molecular interactions regulating cell–cell interactions, we chose Mac/Mono, dominating in cell–cell interactions (Fig. S10B), to map ligand–receptor pair interactions (Fig. S10H). In brief, the “CD74-COPA” ligand–receptor pair was specific in the interactions between Mac/Mono and B cells, Mac/Mono and endothelial cells, particularly in estrogen group. CD74 and its ligands are mainly involved in cell proliferation and monocyte recruitment response [46]. These results are consistent with the role of high levels of estrogen, promoting immune responses in lung tissue and with a worse prognosis [47, 48].

### Estrogen-induced abnormal immune response in breast tissue

**Figure 5.**
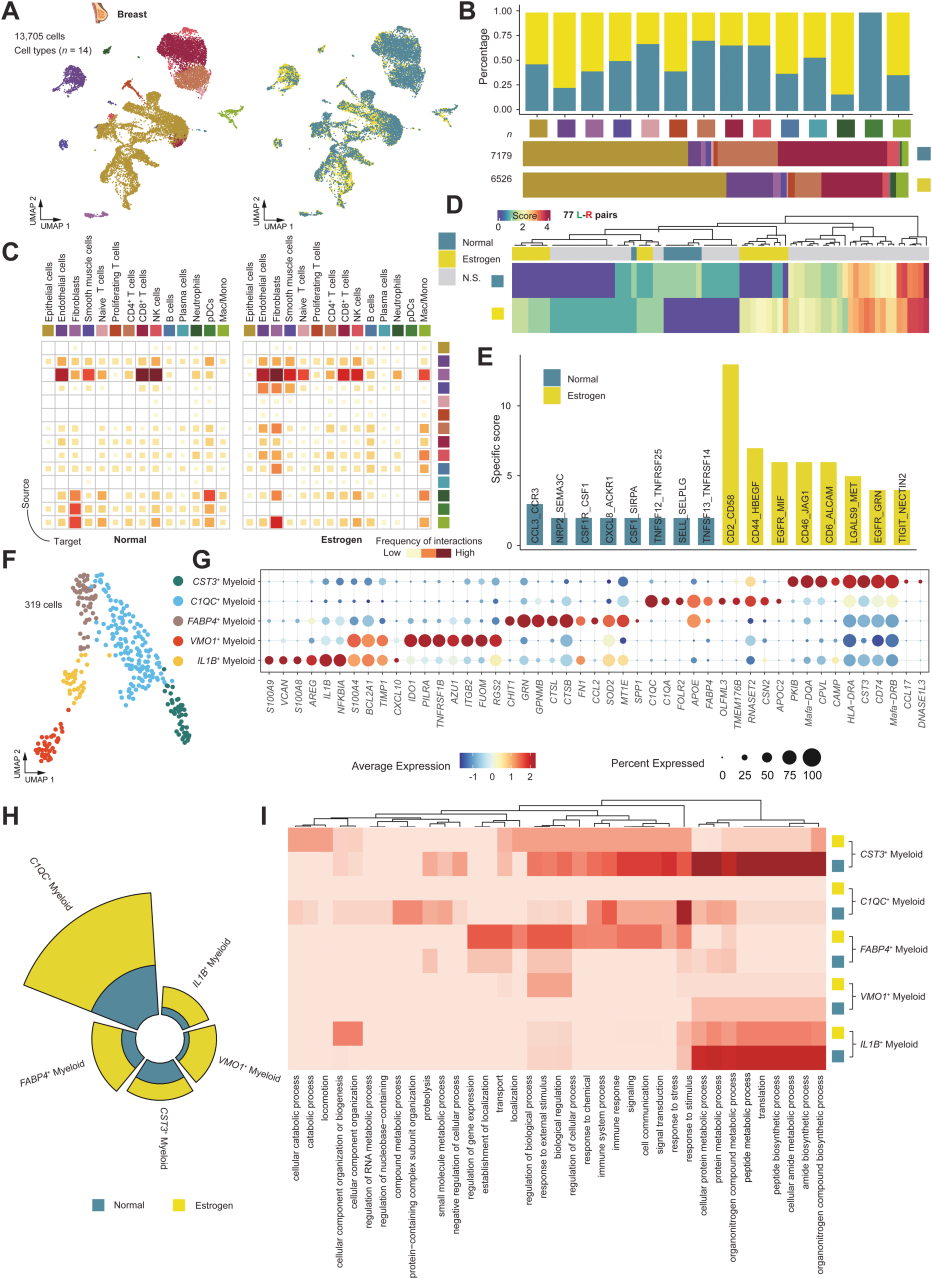
**Estrogen induces an abnormal immune response in breast tissue.**(A) Cell type identification based on scRNA-seq data. In total, 14 major cell types were identified. UMAP visualizations of cells were colored either by the major cell types (left) or by the sample groups (right). (B) Bar plot showing the proportion of breast cell types. Grouped by different cell types (up) and sample groups (down). (C) Heatmap showing the interaction intensity of cellular interactome from the Normal and Estrogen groups. (D) Heatmap showing the interaction scores for 77 ligand–receptor pairs in Normal and Estrogen groups, respectively. Group specificity is displayed on the left of the heatmap. (E) Bar plot showing the top eight group-specific ligand-receptor pairs in two groups. (F) UMAP visualization of the myeloid subclusters. (G) Dot plot showing the representative marker genes across the myeloid subtypes. Dot size is proportional to the fraction of cells expressing specific genes. Color intensity corresponds to the relative expression of specific genes. (H) Nightingale Rose Chart showing the percentage of the Mac/Mono cell subtypes in different sample groups. (I) Heatmap showing the enrichment of functional pathways in myeloid subtypes based on GO pathway enrichment analysis.

Monkey breast tissues yielded 13,705 cells. We identified 14 major cell types using relevant cell markers (Figs. 5A and S11A). Further, 47 subclusters from all cell types were obtained due to specific gene expressions (Fig. S11B), and changes in each of the cell types were additionally observed (Figs. 5B and S11C). Notably, epithelial cells predominate. Then, we found Mac/Mono cells displayed greater intercellular connections (Fig. 5C). Meaningful group specificity was also seen in the interaction scores for 77 ligand–receptor pairs (Fig. 5D). These interactions are crucial for cellular immunity. Of the eight most frequent group-specific pairs in the two groups, the “CD44_HBEGF” pair and the “EGFR_MIF” pair occurred exclusively in the interaction of cells in the estrogen-treated group (Fig. 5E). Additionally, we identified five myeloid subclusters (Fig. 5F and 5G). They differed in proportion and amount changes (Fig. 5H), which was consistent with our GO enrichment analysis, highlighting significant biological pathway variations between clusters and groups (Fig. 5I). Terms enriched indicated that these myeloid-preserved functional adjustments and alterations, after being treated by estrogen, subsequently contributed to the immunological changes. In summary, estrogen orchestrates diverse biological events in the breast, leading to an aberrant immune response. The findings presented herein may provide insights into the potential association between estrogen exposure and an adverse prognosis [49].

### Effects of estrogen on immune microenvironment of liver tissue

**Figure 6.**
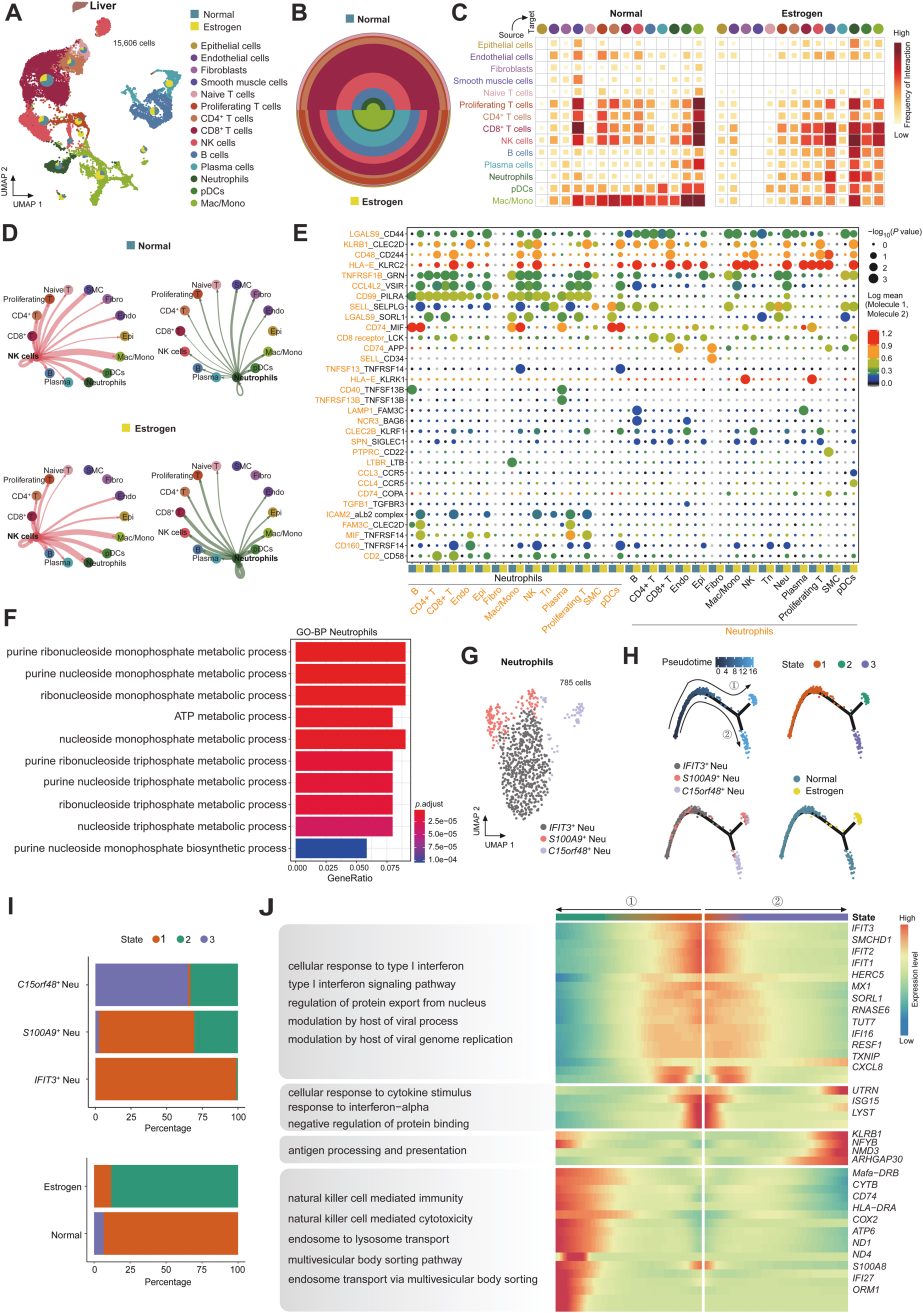
**Effects of estrogen on immune microenvironment of liver tissue.**(A) UMAP plot displaying the integrated cell map of live tissue based on scRNA-seq data, consisting of 15,606 cells from 14 annotated cell types. Cells are colored by cell types. Pie chart showing the proportion of two sample groups, estrogen-treated and normal groups, in each cell types. (B) Pie chart showing the proportion of cell types in two sample groups in the liver. (C) Heatmap showing the cell–cell interaction intensity among liver cell types in two sample groups. Block sizes and colors are proportional to the interaction frequency. (D) Network of NK cells and neutrophils of liver cell types in two sample groups. The thickness represents interaction frequency. (E) Ligand–receptor interactions between neutrophils with all liver cell types. Each row represents a ligand–receptor pair, and each column defines a pair of cell–cell interactions. *P*-values were calculated by CellPhoneDB without multiple comparisons. (F) Barplot showing the top 10 terms of BP enrichment terms for DEGs (estrogen group vs. normal group) of neutrophils. (G) UMAP plot displaying three annotated subtypes map of neutrophils. (H) Pseudotime trajectory analysis of neutrophil subtypes by Monocle2. The trajectory plot indicates pseudotime (top left), cell states (top right), subtypes (bottom left), and sample groups (bottom right). (I) Bar plots showing the distribution of different states of neutrophil subtypes and groups. (J) Heatmap showing the scaled expression of neutrophil DEGs (compared between different neutrophil subtypes) across pseudotime from (H). Genes (listed to the right of the heatmap) are assigned to specific pseudotime based on their expression levels. GO terms to the left of the heatmap represent biological processes following two pseudotime differentiation trajectory.

Many studies have shown that estrogen may have some effects on liver function [50, 51]. Our scRNA-seq analysis of the monkey liver revealed 14 clusters (Fig. 6A). We observed changes in the proportion of various cells after estrogen treatment (Fig. 6B). The interaction of NK cells and neutrophils with other cells was significantly enhanced in the estrogen-treated group compared to the normal group (Fig. 6C and 6D). For example, estrogens significantly induced interactions between neutrophils and epithelial cells, primarily involving the ligand–receptor KLRB1-CLEC2D (Fig. 6E), known as a potential target in immunotherapy [52]. Other interactions among immune cells also underwent varying degrees of alteration (Fig. S12A). In addition, estrogen could induce metabolic process-related pathways in neutrophils of the liver (Fig. 6F). We observed three neutrophil subtypes in the liver, including *IFIT3*^+^ Neu, *S100A9*^+^ Neu, and *C150rf48*^+^ Neu (Fig. 6G). Notably, pseudotime trajectory analysis of the neutrophil population showed that the neutrophils were arranged into a trajectory with two main bifurcations and three cell states (Fig. 6H). We noticed that estrogen -induced the differentiation of *IFIT3*^+^ Neu into *S100A9*^+^ Neu (Fig. 6H and 6I). We also noticed that *S100A9*^+^ Neu-related genes were activated by estrogen (Fig. S12B) and became involved in the function of NK cell-mediated immunity and cytotoxicity, endosome-to-lysosome transport, and the multivesicular body (MVB) sorting pathway (Fig. 6J). Taken together, the functions mentioned above imply that the liver protects from excessive inflammation induced by estrogen. *S100A9*^*+*^ neutrophils interact with NK cells to play key roles during the process.

### Estrogen drives changes in fibroblast function and phenotype in colon tissue

We collected 14,564 monkey colon cells. Based on scRNA data, we identified 14 distinct major cell types (Fig. S13A and S13B). The integrated transcriptomic cell map mostly consists of B cells, T cells, and epithelial cells, with fibroblasts comprising a smaller proportion (Fig. S13C). For intercellular communication dynamics, fibroblasts interacted more strongly with other cells (Fig. S13D and S13E). Furthermore, *FIBIN* and *ADH1B* expression categorized fibroblasts into two populations. *FIBIN*^*+*^ fibroblasts increased significantly after estrogen treatment, while *ADH1B*^*+*^ fibroblasts decreased somewhat (Fig. S13F and S13G). Response to stimulus, structure development, and metabolic process were enriched in these fibroblasts (Fig. S13H). This aligns with the existing understanding that fibroblasts play a vital role in biological processes. Furthermore, we conducted pseudotime trajectory analysis using SCORPIUS to explore their developmental and functional dynamics. The inferred trajectory showed that fibroblasts were aggregated into two cellular states, ordered into the same functional and constitutive group, and clustered independently (Fig. S13I). Dynamic expression changes of the top 3000 genes scaled over pseudotime courses were shown (Fig. S13J), supporting the reliability of the trajectory analysis. Essential genes such as *BATF2*, *ALOX5*, and *Fcgr1*, which would display crucial roles in cell states and identities were observed. Overall, discrete and non-overlapping fibroblast subtypes possess the potential to drive various biological functions. The investigation of phenotypic and functional alterations of fibroblasts may provide insights into the inflammatory context associated with estrogen treatment.

### Transcriptional profiling of uterus stimulated by estrogen

**Figure 7.**
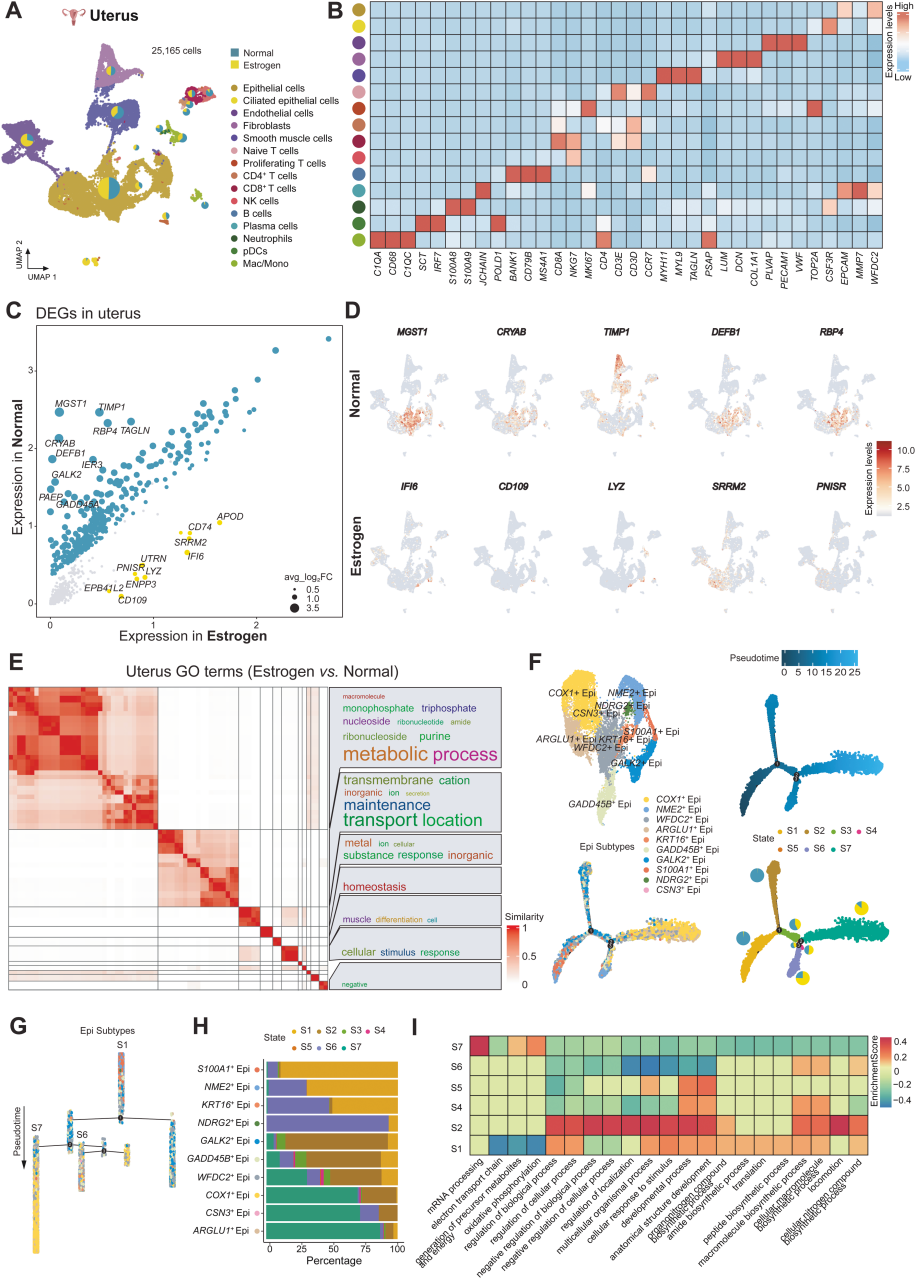
**Transcriptional profiling of uterus stimulated by estrogen.**(A) UMAP plot displaying the integrated cell map of uterus tissue based on scRNA-seq data, consisting of 25,165 cells from 15 annotated cell types. Cells are colored by cell types. Pie chart showing the proportion of two sample groups, estrogen-treated and normal groups, in each cell type. (B) Heatmap showing the expression of cell type-specific gene markers in each cell type in the uterus. (C) Scatter plots showing DEGs between the estrogen-treated and normal group, and the top 10 DEGs are marked. Each dot represents a DEG, and its size is proportional to the fold change. (D) FeaturePlot showing the top five DEGs from (C). (E) SimplifyEnrichment shows significant GO terms of the estrogen-treated group compared with normal group from clustered by “binary_cut” in uterus. (F) UMAP plot displaying 10 annotated subtypes map of epithelial cells (top left). Pseudotime trajectory of epithelial cell subtypes by Monocle2. Trajectory is colored by pseudotime (top right), subtype clusters (bottom left), cell states, and pie plots (bottom right) showing the proportion of sample groups showing on each state. (G) Distribution of epithelial cell subtypes with pseudotime. (H) Bar plots showing the proportion of states (S1–S7) in respective epithelial cell subtypes. (I) Heatmap showing the functional pathways both highly and poorly enriched in six cell states (S1–S2, S4–S7) of epithelial cells by GSEA. (S3 has no enrichment pathway.) *P* value < 0.01.

It should be noted that estrogen mostly affects the uterus [13]. In this section, we identified 15 clusters in uterus tissues based on their maker genes (Fig. 7A and 7B). Epithelial cells account for approximately half of the total cell population. (Fig. S14A). Next, we identified several DEGs between the normal group including *MGST1*, *CRYAB*, and *TIMP1* and the estrogen group including *IF16*, *CD109*, and *LYZ*, etc (Fig. 7C). Interestingly, most of these DEGs were expressed in epithelial cells (Figs. 7D and S14B), suggesting that estrogen may mainly affect the function of epithelial cells. GO enrichment analysis (Fig. 7E) revealed that the estrogen group was largely involved in the metabolic process. However, transport location and response to inorganic substances were significantly down-regulated (Figs. 7E and S14C). To dissect epithelial cells’ heterogeneity, we identified 10 epithelial cell subtypes (Fig. 7F) and performed pseudotime trajectory analysis. Epithelial cells were spliced into seven states. The predominant cell subtypes at the initiation point of differentiation were *S100A1*^+^ Epi, existing primarily in State1, and *COX1*^+^ Epi and *ARGLU1*^+^ Epi in State7 were at the end point (Fig. 7F–H). It was evident that estrogen-treated epithelial cells were mainly clustered in the terminal differentiation position, State7, implying that *COX1*^+^ Epi and *ARGLU1*^*+*^ Epi played specific functions after estrogen stimulation, which could also be concluded from Fig. S14D. In State7, mRNA processing and oxidative phosphorylation were highly up-regulated (Fig. 7I). These functions are closely related to mitochondria. And *COX1*^*+*^ Epi highly expressed mitochondrial genes as well, such as *COX1*, *ND4*, and *ND5*. To explore *COX1*^*+*^ Epi’s biological function in more detail, we performed GO enrichment analysis of it compared to other epithelial cell subtypes. *COX1*^*+*^ Epi focused on metabolic process (Fig. S14E). The similarity to GO analysis of the whole uterus tissue (Fig. 7E) highlighted the main position of *COX1*^*+*^ Epi under estrogen treatment. The results above suggest that estrogen significantly upregulates the expression and functions of mitochondrial genes in the uterus.

## Discussion

Long-term high levels of estrogen in the body often cause an imbalance in the body’s related functions [19, 53]. Although several studies have recently established the relationship between abnormal estrogen levels and diseases [54, 55], an organism-wide single-cell map to explore the role of estrogen is still lacking. Here, we charted a reference cell map of cynomolgus monkeys across multiple organs, allowing deeper insights into the effect of estrogen on molecular dynamics and cellular heterogeneity of the cynomolgus monkeys’ organism. The analysis results of lung, breast, liver, colon, and uterus show that estrogen is more like a pro-inflammatory factor, causing sterile inflammation and inducing immune responses in various organs.

To explore the estrogen-mediated regulatory network, we applied SCENIC [56] to identify TF regulons based on co-expression and motif enrichment. We observed that regulons have certain conformance in the same cell types even in different organs. In addition, we identified specific TFs driven by estrogen in specific organs. For example, target genes of ETV2 and POGK, which are associated with cell differentiation and tumor angiogenesis, were specifically expressed in the uterus from the estrogen group [25, 57, 58]. We also observed that SOX13 was found to be a specific regulon in uterine epithelial cells in the estrogen group. Notably, the function of *SOX13* is associated with cancer development [59]. This is consistent with the traditional view that chronically high levels of estrogen are a risk factor for endometrial hyperplasia and endometrial cancer development. Many studies have reported the effect of estrogen on body homeostasis [60]. However, how estrogen is involved in disease progression has been relatively poorly studied. To solve this problem, we integrated our datasets with the NHGRI-EBI GWAS database [61]. We found that high levels of estrogen-induced abnormal expression of many disease-risk genes. For example, we identified that several genes, such as *BPTF* and *MDN1*, were upregulated in uterine epithelial cells after estrogen treatment. These genes are related to endometrial cancer development. This is consistent with the traditional view that long-term high levels of estrogen can cause endometrial hyperplasia and endometrial cancer [62, 63]. Many studies support that estrogen can inhibit colon cancer progression [64, 65]. However, several studies reported that estrogen contributes to the late stage of colon cancer [66]. In our data, we noticed that estrogen suppressed *RPS21* expression but increased *HSP11* expression in almost all cell types. Both *RPS21* and *HSPH1* have been reported to be involved in tumor progression. This partly explains why estrogen plays a double-sided role in colon cancer progression. Also, this may depend on the stage of the disease and the gender of the patient. Furthermore, we found that the genes mediated by high levels of estrogen highly overlapped with the risk genes for COVID-19 and asthma diseases, involving *HLA-DRA*, *HLA-F*, and *HLA-DOB*. These genes play an important role in the immune response [40, 41]. Interestingly, we noticed that estrogen-induced abnormal gene expression was almost not involved in liver disease risk genes. Overall, we found that high levels of estrogen significantly induced the expression of disease-related risk genes in the uterus, lung, and breast. On the one hand, estrogen can inhibit the expression of tumor-promoting genes, and on the other hand, it can promote the expression of other oncogenes in colon tissue. These results may help us understand how estrogen induces or protects against disease progression.

Considering the different cell compositions of each organ, we analyzed the effect of estrogen on each organ. Many studies have reported the effect of estrogen on immune function [67, 68]. According to the results of our analysis, estrogen mainly affects the changes in immune microenvironment in the liver, breast, and lung tissues. For example, estrogen drives the interaction of “CD74-COPA” ligand–receptor pairs between immune cells, especially among Mac/Mono, B cells, and endothelial cells in the lung tissue. It is worth mentioning that the enhanced interaction of the “CD74-COPA” ligand pair has been shown to mediate immune disorders and thus promote the progression of multiple tumor diseases [69, 70]. In breast tissues, we noticed estrogen significantly enhanced the function of immune system processes and the immune response of myeloid cells. According to previous reports, high levels of estrogen increase the risk of breast cancer [71, 72], which may be related to the abnormal function of myeloid cells driven by estrogen. Among immune cells in the liver tissues, the interaction of neutrophils with other cells was significantly enhanced in the estrogen-treated group compared to the normal group. We observed three neutrophil subtypes in the liver and found that estrogen-induced the differentiation of *IFIT3*^+^ Neu into *S100A9*^+^ Neu. In this process, we noticed that *S100A9*^+^ Neu-related genes were activated by estrogen and became involved in the function of endosome-to-lysosome transport and the multivesicular body sorting pathway. This suggests that estrogen may increase the risk of disease by mediating the differentiation of neutrophil subsets in liver tissue. It is also notable that estrogen showed more effects on tissue cells in the uterus and colon. For example, the function of mRNA processing and oxidative phosphorylation were highly up-regulated in *COX1*^+^ epithelial cells from the estrogen-treated group. Following estrogen application, there was a significant increase in the population of the *FIBIN*^*+*^ fibroblasts, while the *ADH1B*^*+*^ fibroblasts exhibited a little reduction in abundance. These results indicated that estrogen drives changes in fibroblast function and phenotype in colon tissue. Overall, estrogen mediates multiple biological events. It is important to note that the effector cells of estrogen are different in different tissues. In addition, estrogen regulates different signaling pathways in different cells.

**Figure 8.**
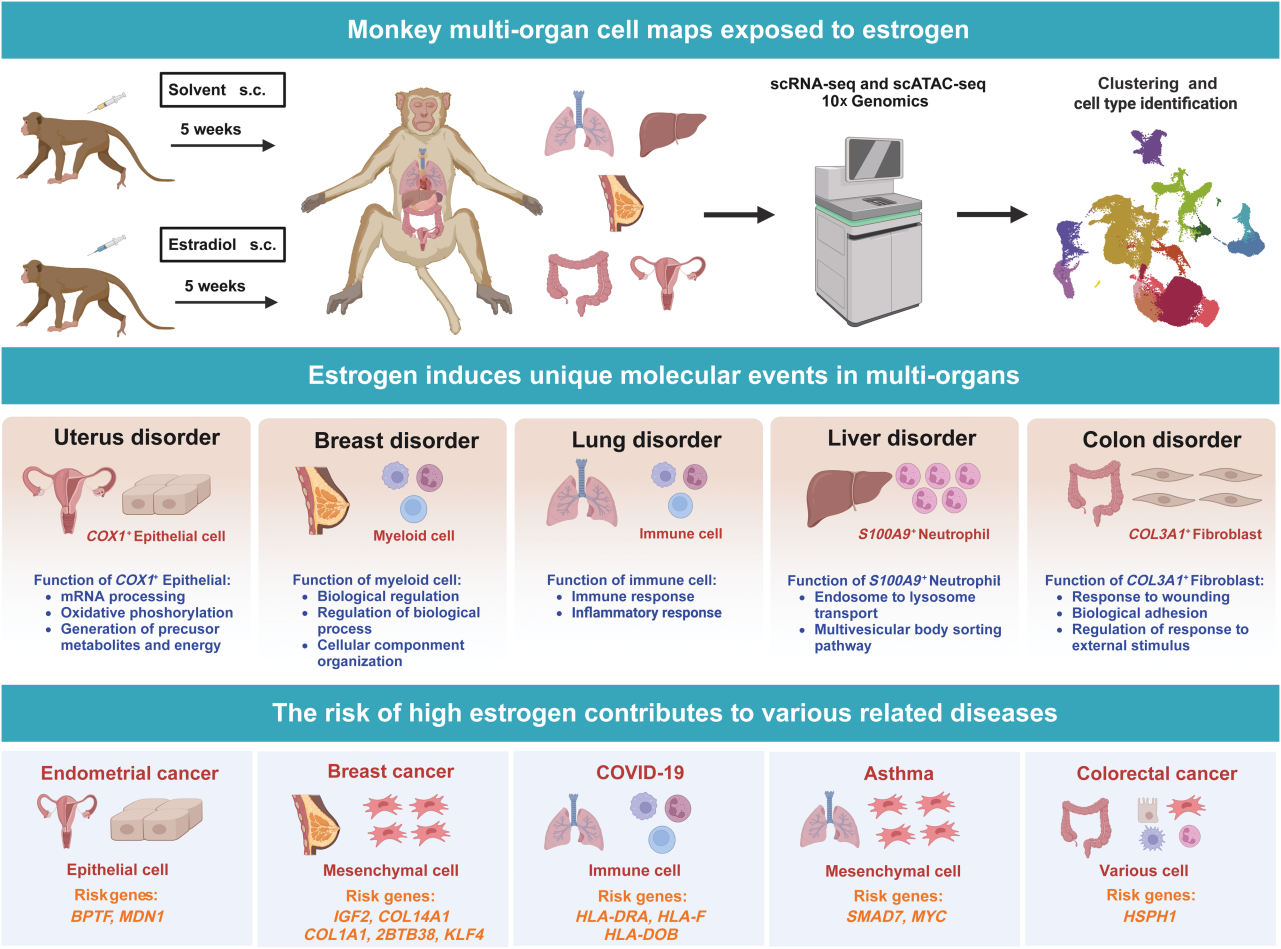
**Graphical summary.**The significant and innovative features of this study are summarized below: (1) Our work highlights the impact of estrogen on the major organs in monkeys, which constitutes a useful resource for many scientists. (2) Using single-cell transcriptomic data, we infer pseudotime cell trajectories and cell-cell communications to uncover key molecular signatures underlying associated cellular processes in major organs after estrogen stimulation. (3) We integrate our datasets with the NHGRI-EBI GWAS database and identify a subset of risk genes during disease development that are induced by estrogen.

In conclusion, the integrated cell map enables in-depth dissection and comparison of molecular dynamics, cell-type compositions, and cellular heterogeneity across multiple tissues and organs under estrogen stimulation. This study provides an extensive resource of estrogen-driven changes and identifies genes that potentiate the risk of estrogen-associated disorders (Fig. 8).

## Research limitations

Although we have provided relatively rich single-cell multi-omics data in the cynomolgus monkey, there are still several limitations to the current study. There are still many functionally important organs (such as the ovary, pancreas, and cerebellum) that have not yet been included in our study due to limited resources. In addition, some scRNA-seq samples do not have matched scATAC-seq data, which may restrict unbiased exploration of DNA regulatory elements in specific organs. Some studies have also confirmed the potential effects of estrogen on males [73–75]. However, we only studied the effects of estrogen on the vital organs of female cynomolgus monkeys. The effect of estrogen on male vital organs is worthy of further investigation. Moreover, we only focused on the effect of estrogen on multiple organs under physiological conditions and did not explore the effect of estrogen under the background of disease.

## Methods

### Research ethics

Healthy female cynomolgus monkeys ranging from 3 to 5 years of age were raised in the monkey breeding base of Changchun Biotechnology Development Co., Ltd., Guangxi, China. The sample collection of cynomolgus monkeys and research conduction in this study was approved by the Research Ethics Committee of the Changchun Biotechnology Development Co., Ltd. (Approval Number: 200010). The managing protocols of the monkeys were carried out in accordance with the standard procedures of Changchun Biotechnology Development, referring to the Guide for Care and Use of Laboratory Animals (2010) and the principles of Animal Welfare Management (Public Law 99-198).

### Organ tissue collection of monkeys

The monkeys were coded and randomly divided into two groups. Estradiol (32 μg/kg) was s.c. injected into cynomolgus monkeys for five successive weeks. The control group was given the same amount of solvent induction. After the experiment, the tissue was collected for follow-up experiments.

### Tissue collection

Tissues were collected from monkeys and tissue digestion was performed according to the manufacturer’s protocol. Single cells were captured by the 10× Genomics Chromium Single Cell 3ʹ Solution. scRNA-seq library was conducted by Shanghai Xuran Biotechnology and scATAC-seq library was conducted by LC-Biotechnology (Hangzhou, China) and prepared following the manufacturer’s protocol (10× Genomics). The libraries were subjected to high-throughput sequencing on the Novaseq6000 platform, and 150-bp paired-end reads were generated.

### scRNA-seq and data processing

The reference genome sequence of *Macaca fascicularis* in FASTA format and gene annotation in GTF format were downloaded from the ENSEMBL database. Raw scRNA-seq data were aligned to the *M. fascicularis* reference genome (macFas6) [76], and subjected to barcode assignment and unique molecular identifier (UMI) counting using the CellRanger (v3.1.0) pipeline (10× Genomics). Filtered count matrices from the CellRanger pipeline were converted to sparse matrices using the Seurat package (v4.0.0) [77]. Potential doublets were detected and filtered using DoubletFinder based on the expression proximity of each cell to artificial doublets [78]. Cells that expressed either more than 4000 genes or less than 200 genes, as well as those with more than 20% mitochondrial gene expression in UMI counts were removed from the analysis. Filtered data were then log normalized and scaled to avoid cell-to-cell variation caused by UMI counts and the percent mitochondrial reads. Specifically, the top 3000 most variably expressed genes were determined using the “vst” method in the “FindVariableFeatures” function and scaled using “ScaleData” with regression on the proportion of mitochondrial UMIs (mt.percent). We used the RPCA method in Seurat for the integration of scRNA-seq data from different organs. The “RunPCA” function was used to compute the top 20 principal components using variably expressed genes. We used UMAP for the visualization of cell clusters. Clustering was performed for integrated expression values based on shared-nearest-neighbor graph clustering (Louvain community detection-based method) using “FindClusters” with a resolution of 0.5. We used the “FindAllMarkers” function with default parameters to identify markers for each cluster.

### Id*entification of signature genes*

We applied the “FindAllMarkers” function in Seurat to identify specific genes for each cell subset. For the selection of marker genes specific to each cell cluster/subset, we calculated the log_2_ fold change (log_2_FC) between two groups (a cell cluster/subset *vs.* other cells) using the “FindMarkers” function with the Wilcoxon rank-sum test (default parameters).

### Identification of MPs that collectively respond to the estrogen signaling within each organ across various major cell types

For all major cell types, we separately used a non-negative factorization algorithm (documented by the python cNMF package) [79] to identify underlying intra-organ expression programs within the five organ samples with estrogen-treated. For each sample, we applied NMF to the relative expression matrix with all negative values replaced by zero. We filtered genes with standard deviations of expression < 0.5 within each sample. We selected different values of *k* as the number of factors because it yielded a high cophenetic correlation coefficient and effectively decomposed the dataset of each sample. We listed the top-ranked genes according to their loadings of the NMF factor. All metagenes were then compared by hierarchical clustering, using one minus the Pearson correlation coefficient over all gene scores as a distance metric. For each signature, we then combined the top 100 genes of each metagene and calculated the average loadings for each gene [80]. We summarized the total loadings for repetitive genes, retained the original loadings for exclusive genes, and divided the loadings of each gene by the number of metagenes within the signature. Finally, the top 30 genes with the highest loading were defined as the marker genes for the signature.

### Create a cisTarget database for M. fascicularis

We followed the instruction by SCENIC (github.com/aertslab/create_cisTarget_databases) [81] to construct cisTarget database. Since there are no well annotated TF motifs in *M. fascicularis*, we instead used the annotated human motifs from the CIS-BP website (cisbp.ccbr.utoronto.ca/) to create cisTarget databases for *M. fascicularis*.

### Gene-regulatory network

To identify cell-type and organ-specific gene regulatory networks, we performed Single-cell Regulatory Network Inference and Clustering (v0.11.2; a Python implementation of SCENIC) in our *M. fascicularis* dataset. Firstly, the original expression data were normalized by dividing the gene count for each cell by the total number of cells in that cell and multiplying by 10,000, followed by a log1p transformation. Next, normalized counts were used to generate the co-expression module with the GRNboost2 algorithm implemented in the arboreto package (v0.1.6). Finally, we used pySCENIC with its default parameters to infer co-expression modules using the above-created RcisTarget database. An AUCell value matrix was generated to represent the activity of regulators in each cell. Gene regulatory networks (GRNs) were visualized by the igraph package in R.

### Integration of GWAS data

GWAS data were searched and downloaded from the NHGRI-EBI Catalog of human GWASs (*61*). This database contains all human GWASs that meet the NHGRI-EBI quality criteria (more than 100,000 single-nucleotide polymorphisms (SNPs) analyzed in the study). Human genetic variants showing statistically significant association (*P* < 10^−5^) with the indicated disease were compared to DEGs in our scRNA-seq dataset. Child trait data were included. For dot plots comparing GWAS SNP-associated genes and DEGs in our dataset, we used the log_2_ (FC) of the estrogen group versus the normal group and plotted it against the  − log_10_ (*P*-value) of the disease-associated SNP of that gene.

### Pathway analysis

Gene-set enrichment analysis on DEGs in this study was performed by the clusterProfiler [82] package in R. Gene-set variation analysis (GSVA) was conducted using the GSVA package [83]. Expression differences between different cell groups were calculated by the “FindAllMarkers” function in the Seurat package.

### Trajectory inference using Monocle

Monocle2 (v2.99.3) [84] was used to infer the state transition of neutrophils in the liver and epithelial cells in the uterus. Variable genes identified among cell subsets were used to sort cells in the pseudotime analysis. Proliferation scores were based on the scaled mean of expression of proliferation-associated genes, including *KRT7*, *BTF3*, *RACK1*, and *NAP1L1*.

### Trajectory inference using SCORPIU

SCORPIU (version 1.0.9) [85] was chosen for investigation of the fibroblasts in colon. Dimensionality reduction was based on the spearman distance metric.

### Cell–cell interaction analysis

Cell–cell interactions among different cell types were estimated by CellPhoneDB (v2.1.7) [86] with default parameters (10% of cells expressing the ligand/receptor). In order to run CellPhoneDB analysis in cynomolgus monkeys, the *M. fascicularis* genes were converted to human genes based on homologous gene mapping. Interactions with *P*-value < 0.05 were considered to be significant. We considered only ligand–receptor interactions based on the annotation from the CellPhoneDB database and discarded receptor–receptor and other interactions without a clear receptor.

### scATAC-seq data pre-processing

The scATAC-seq sequencing data were pre-processed by cellranger-atac (v1.2.0). Subsequent scATAC-seq data analysis was performed by ArchR (v1.0.1) [87]. Specifically, the *M. fascicularis* genome was constructed and annotated by createGenomeAnnotation and createGeneAnnotation function, respectively. Then arrow file was created by createArrowFiles function with default parameters. We used the addDoubletScores function to infer potential doublets, and the filterDoublets function was used to remove the potential doublets with the “filterRatio = 1.0” parameter. ArchR project was created by ArchRProject function with default parameters. For dimensionality reduction, we used the addIterativeLSI function in ArchR. Next, the Harmony method was utilized to remove the batch effect by the addHarmony function [88]. AddClusters function was used to cluster cells by its default parameters. For single-cell embedding, we selected the reducedDims object with harmony and used addUMAP function with the parameter “nNeighbors = 40, minDist = 0.4” for visualization.

### *Marker genes* id*entification and cluster annotation*

To identify the marker gene, gene scores were calculated when the ArchR project was created and stored in the arrow file. Then getMarkerFeatures function was used to identify the cluster-specific “expressed” genes with default parameters. To visualize the marker genes in the embedding, we used addImputeWeights function to run the MAGIC [89] to smooth gene scores across the nearby cells.

### Peak calling analysis

Before peak calling, we used the addGroupCoverages function with default parameters to make pseudo-bulk replicates. Then the addReproduciblePeakSet function was used with its default parameters except for “genomeSize = 2.9e09” to call accessible chromatin peaks using MACS2 (v2.2.7.1) [90]. For cell-type-specific peak analysis, the getMarkerFeatures function was firstly applied to identify marker peaks. Then the getMarkers function with the parameter “cutOff = FDR < = 0.05 & Log2FC > = 0.25” was conducted to get the differential peaks.

### Integrative analysis of scRNA-seq and scATAC-seq data

In order to integrate scATAC-seq data from different organs, we extracted and annotated the scRNA-seq data with matched scATAC-seq data in the same organ. We first used the FindTransferAnchors function from the Seurat package and aligned the data with the addGeneIntegrationMatrix function in ArchR with “unconstrained integration” mode. To improve the accuracy of the prediction and to better integrate the two datasets, a “constrained integration” mode was applied to integrate the scATAC-seq and scRNA-seq data. Briefly, we annotated the scATAC-seq data with cell types based on the gene scores of scATAC-seq. Then, a restricted list was created to make sure that gene expression similarity was calculated only in the same cell type for both scATAC-seq and scRNA-seq data.

### Statistical and reproducibility

If not specified, all statistical analysis and data visualization were done in R (version 4.0.0). We state that no statistical method was used to predetermine sample size. No data were excluded from the analysis and the experiments were not randomized. The investigators were not blinded to allocation during experiments and outcome assessment.

## Supplementary Material

lnae012_suppl_Supplementary_Figs_S1

lnae012_suppl_Supplementary_Figs_S2

lnae012_suppl_Supplementary_Figs_S3

lnae012_suppl_Supplementary_Figs_S4

lnae012_suppl_Supplementary_Figs_S5

lnae012_suppl_Supplementary_Figs_S6

lnae012_suppl_Supplementary_Figs_S7

lnae012_suppl_Supplementary_Figs_S8

lnae012_suppl_Supplementary_Figs_S9

lnae012_suppl_Supplementary_Figs_S10

lnae012_suppl_Supplementary_Figs_S11

lnae012_suppl_Supplementary_Figs_S12

lnae012_suppl_Supplementary_Figs_S13

lnae012_suppl_Supplementary_Figs_S14

## Data Availability

The data that supports the findings of this study has been uploaded to Gene Expression Omnibus (GEO) with accession number GSE223639 (Normal, www.ncbi.nlm.nih.gov/geo/query/acc.cgi?acc=GSE196792) and GSE243771 (Estrogen, www.ncbi.nlm.nih.gov/geo/query/acc.cgi?acc=GSE243771).
