# Supplementary material for: Monkey multi-organ cell atlas exposed to estrogen
Source: Life Med. 2024 Mar 22;3(2):lnae012. doi: 10.1093/lifemedi/lnae012 (PMC11749546; doi:10.1093/lifemedi/lnae012)
Supplement: lnae012_suppl_Supplementary_Figs_S1 [file lnae012_suppl_Supplementary_Figs_S1.pdf]

Supplementary Figures

Monkey Multi-Organ Cell Atlas Exposed to Estrogen

Wen Fang[1,#], Jiao Qu[1,2,#], Wanjun Zhao[1,#], Xinran Cao[1,#], Jinran Liu[1], Quan Han[1], Dijun Chen[1], Wen Lv[3,*], Yicheng Xie[4,*], Yang Sun[1,2,*]

[1]State Key Laboratory of Pharmaceutical Biotechnology, School of Life Sciences, Nanjing University, Nanjing 210023, China

[2]Jiangsu Key Laboratory of New Drug Research and Clinical Pharmacy, Xuzhou Medical University, Xuzhou 221004, China

[3]Department of Gynecology, Tongde Hospital of Zhejiang Province, 234 Gucui Road, Hangzhou 310012 Zhejiang, China

[4]The Children's Hospital, Zhejiang University School of Medicine, National Clinical Research Center for Child Health, 310052 Hangzhou, China

#These authors contributed equally to this work.
*Correspondence: wl4021@163.com (W.L.), ycxie@zju.edu.cn (Y.X.), yangsun@nju.edu.cn (Y.S.)

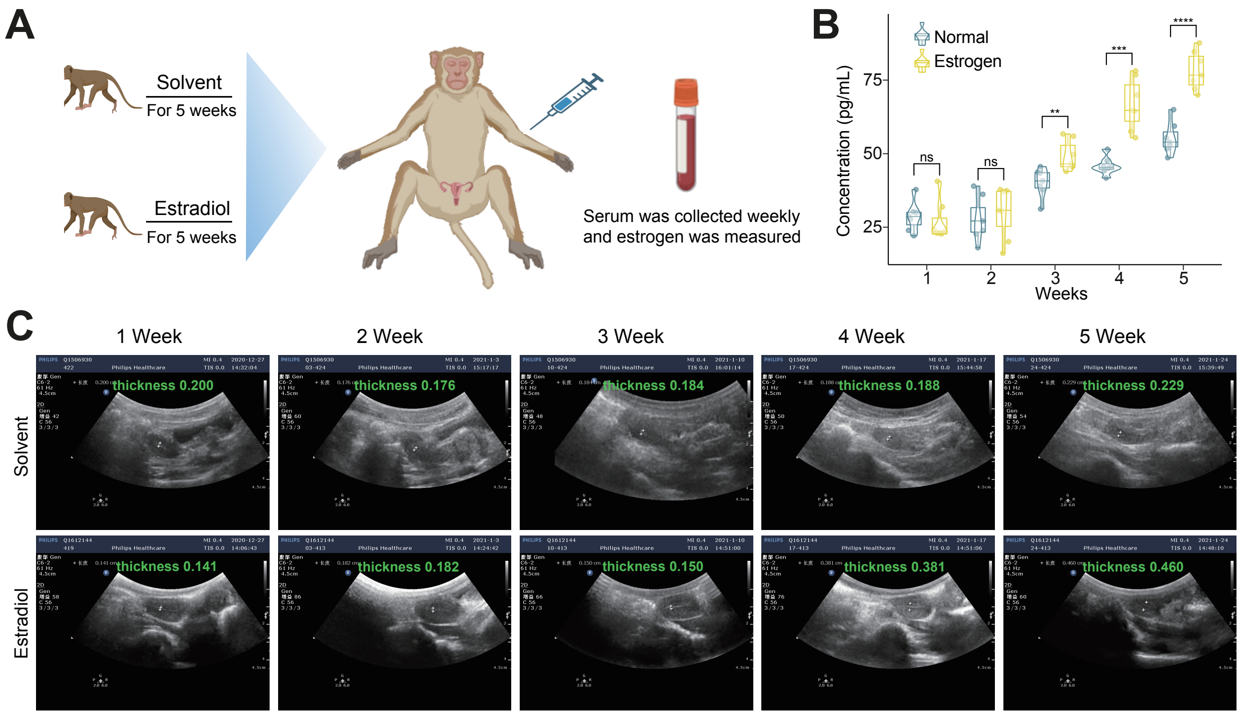

Supplementary Figure 1. Estrogen levels increased after 5 weeks of estradiol induction. Related to Figure 1. (A) Female cynomolgus monkeys were subcutaneously injected with 32 μg/kg estradiol per day for 5 weeks. **(B)** The level of estradiol in the serum of each monkey was determined (right, $n = 7$). The p-values are calculated using two-sided t test. *, $P < 0.05$; **, $P < 0.01$; ***, $P < 0.001$; ****, $P <$ 1e-04. The boxes indicate the median (horizontal line), second to third quartiles (box), and Tukey-style whiskers (beyond the box). **(C)** Abdominal ultrasound of the cynomolgus monkey reveals the endometrial thickness of the cynomolgus monkey in different weeks (cm).
