# Supplementary material for: Monkey multi-organ cell atlas exposed to estrogen
Source: Life Med. 2024 Mar 22;3(2):lnae012. doi: 10.1093/lifemedi/lnae012 (PMC11749546; doi:10.1093/lifemedi/lnae012)
Supplement: lnae012_suppl_Supplementary_Figs_S2 [file lnae012_suppl_Supplementary_Figs_S2.pdf]

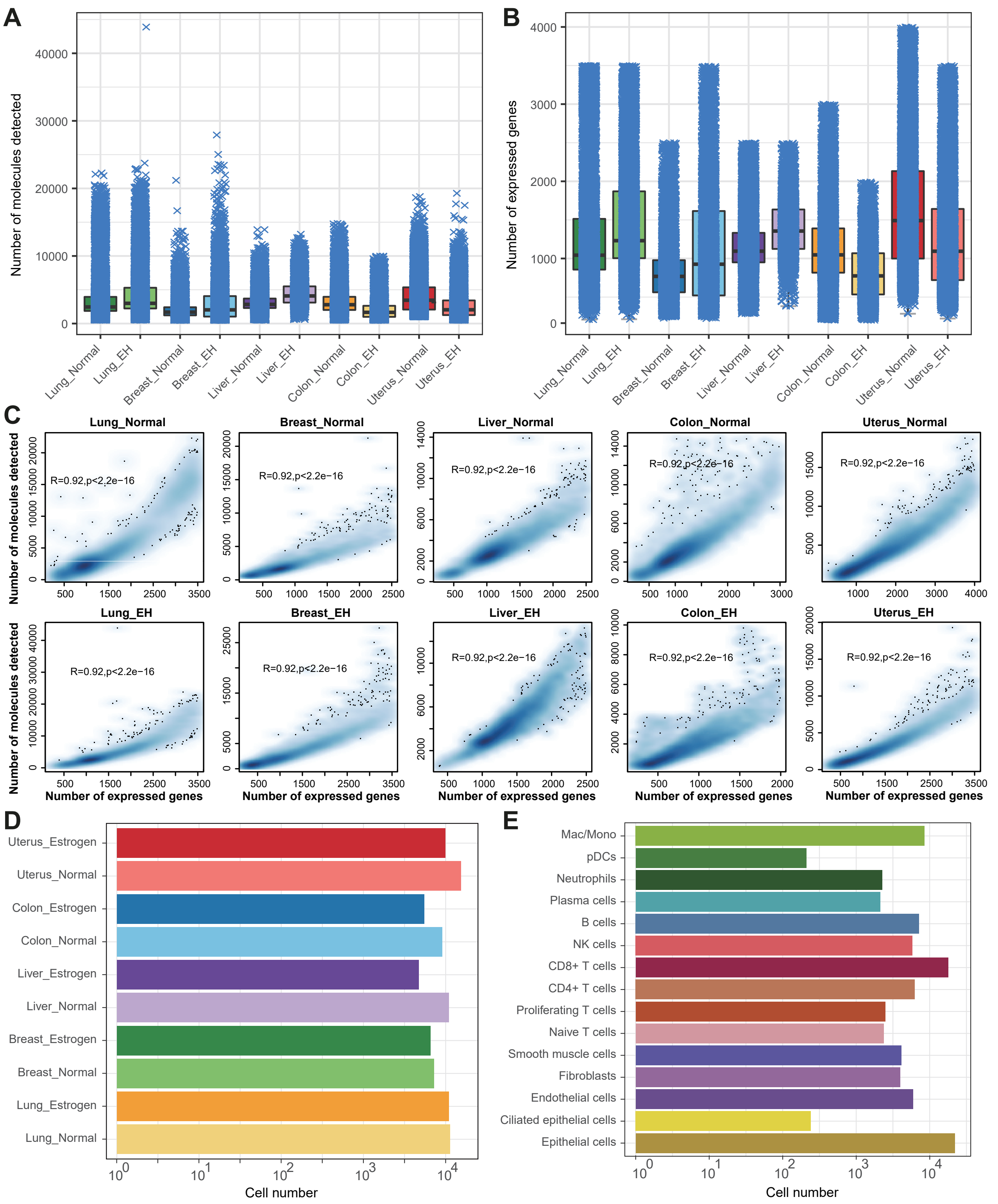

Supplementary Figure 2. Quality control (QC) of scRNA-seq data. Related to Figure 1. (A-B) Box plot showing the number of UMIs and genes in samples, respectively. The boxes indicate the 25% quantile, median (horizontal line), and 75% quantile. **(C)** Positive correlation between the number of gene and UMI in the cells of each sample. Correlations were calculated using pearson. **(D-E)** Bar plots showing the number of cells in samples and in different cell types.
