# Supplementary material for: Monkey multi-organ cell atlas exposed to estrogen
Source: Life Med. 2024 Mar 22;3(2):lnae012. doi: 10.1093/lifemedi/lnae012 (PMC11749546; doi:10.1093/lifemedi/lnae012)
Supplement: lnae012_suppl_Supplementary_Figs_S3 [file lnae012_suppl_Supplementary_Figs_S3.pdf]

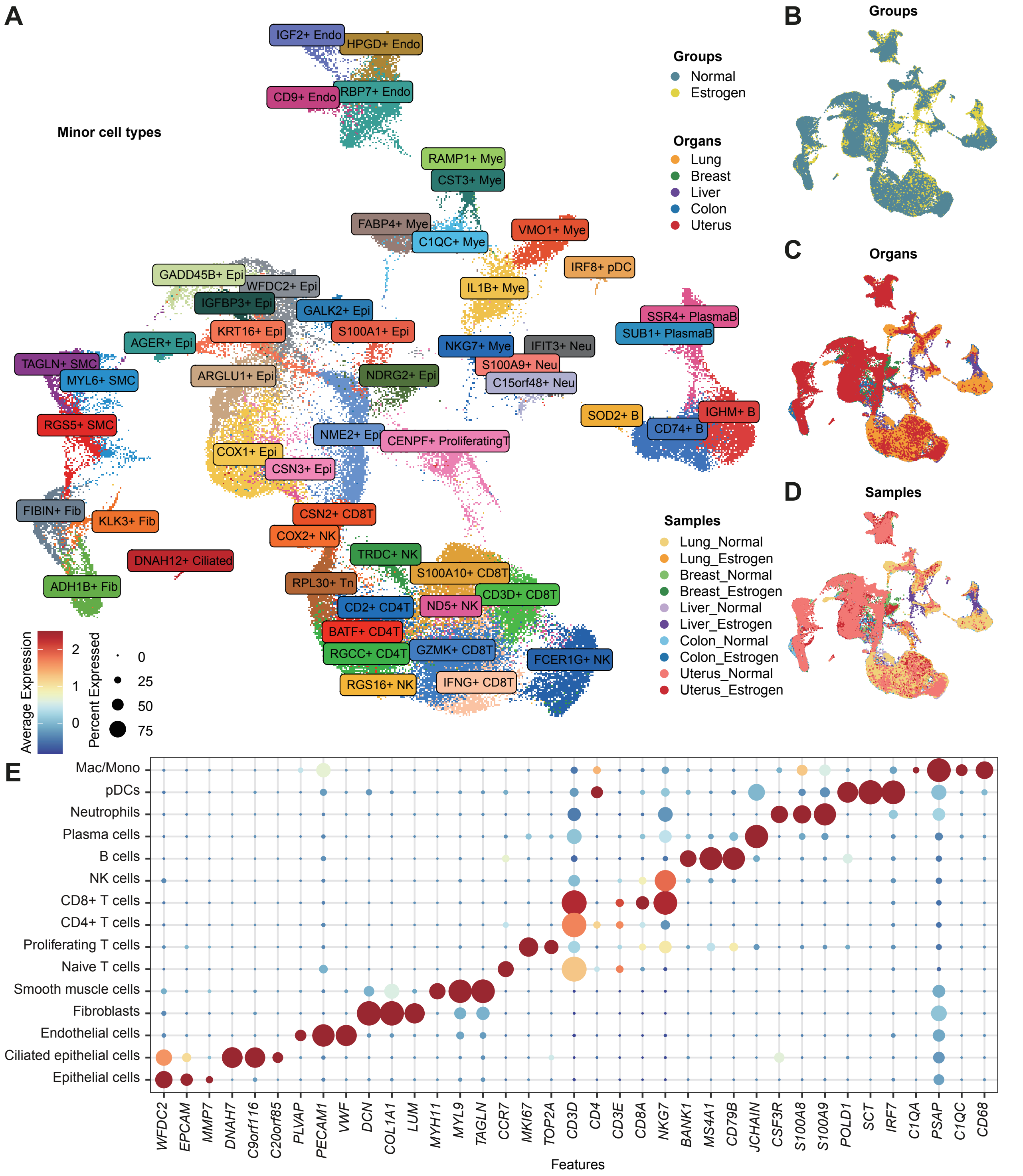

Supplementary Figure 3. Cell clustering and cell type annotation based on UMAP. Related to Figure 1. (A) UMAP showing the distribution of minor cell types. Cells are colored by minor cell types. **(B)** UMAP showing the distribution of groups. Cells are colored by groups. **(C)** UMAP showing the distribution of organs. Cells are colored by organs. **(D)** UMAP showing the distribution of samples. Cells are colored by samples. **(E)** Dot plot showing representative marker genes across cell clusters. Dot size is proportional to the fraction of cells expressing specific genes. Color intensity corresponds to the relative expression of specific genes.
