# Supplementary material for: Monkey multi-organ cell atlas exposed to estrogen
Source: Life Med. 2024 Mar 22;3(2):lnae012. doi: 10.1093/lifemedi/lnae012 (PMC11749546; doi:10.1093/lifemedi/lnae012)
Supplement: lnae012_suppl_Supplementary_Figs_S4 [file lnae012_suppl_Supplementary_Figs_S4.pdf]

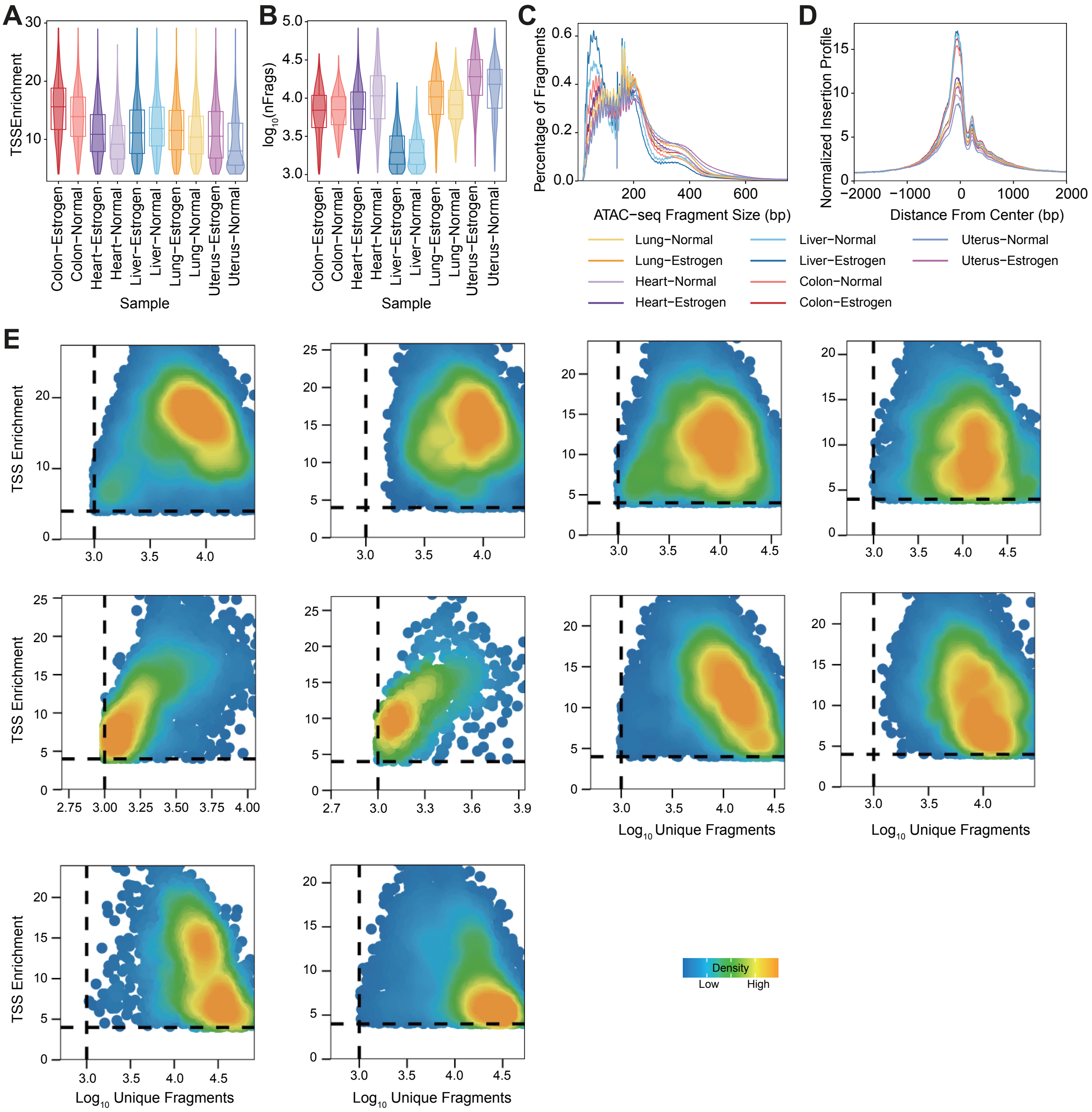

Supplementary Figure 4. Quality control of scATAC-seq data. Related to Figure 1. (A-B) Box plot showing the distribution of the TSS enrichment score and the number of fragments across eight samples. Each dot represents a cell. The boxes indicate the 25% quantile, median (horizontal line), 75% quantile, and Tukey-style whiskers (beyond the box). **(C-D)** Fragment size distributions of eight samples (left). Aggregate TSS insertion profiles are centred at all TSS regions. The showing cells are passing ArchR QC thresholds for each sample (right). **(E)** Scatter plot showing the TSS enrichment score vs. unique nuclear fragments per cell. The colour of the dots represents the density of each point in the plot.
