# Supplementary material for: Monkey multi-organ cell atlas exposed to estrogen
Source: Life Med. 2024 Mar 22;3(2):lnae012. doi: 10.1093/lifemedi/lnae012 (PMC11749546; doi:10.1093/lifemedi/lnae012)
Supplement: lnae012_suppl_Supplementary_Figs_S5 [file lnae012_suppl_Supplementary_Figs_S5.pdf]

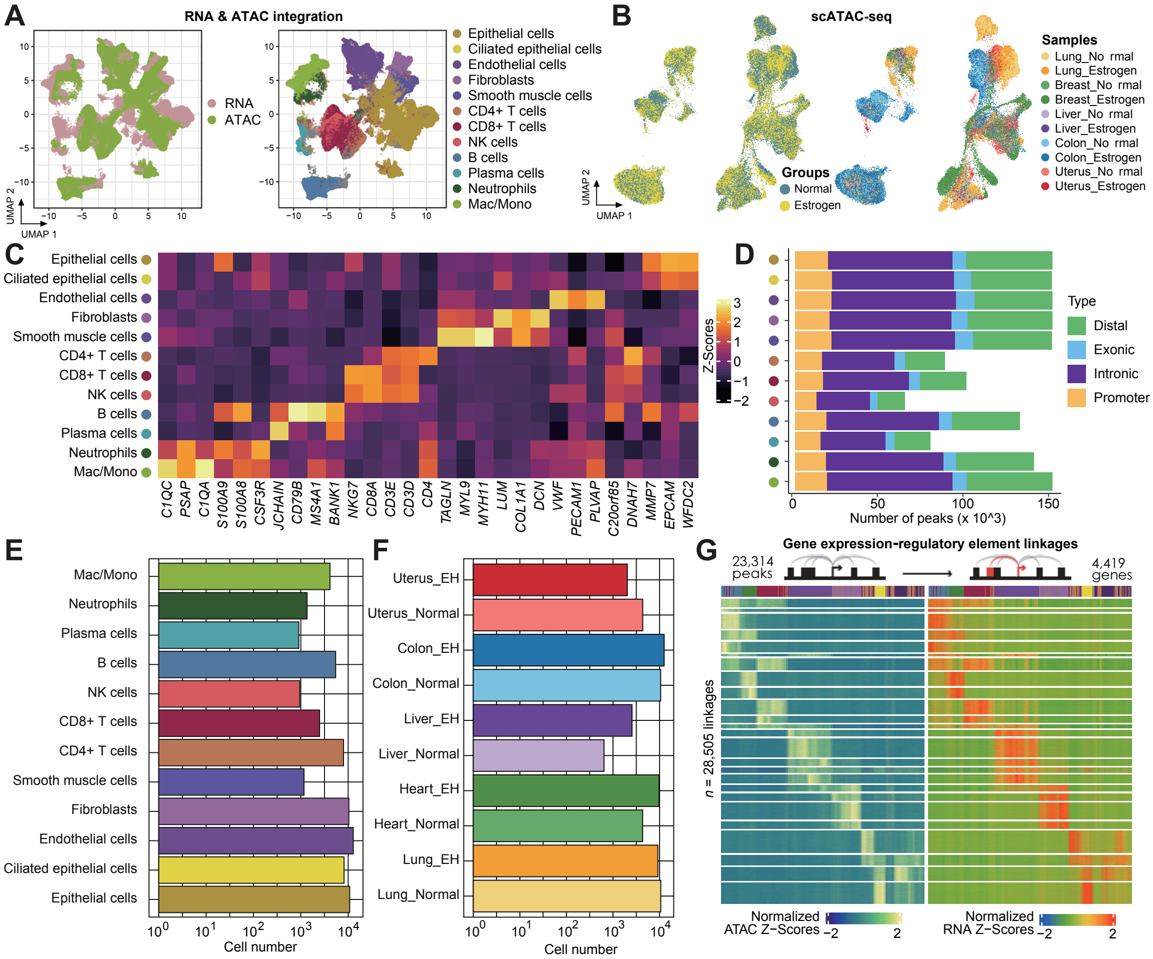

Supplementary Figure 5. Integrative analysis of scRNA-seq and scATAC-seq data. Related to Figure 1. (A) UMAP plot showing the joint clustering of scRNA-seq (red) and scATAC-seq (blue) data. Cells in the right UMAP are colored by cell types. **(B)** UMAP showing the distribution of groups (left) and samples (right). **(C)** Heatmap showing gene-activity scores of marker genes in the indicated cell types. **(D)** Bar plot showing the number of reproducible peaks identified from each cluster. **(E-F)** Bar plots showing the number of cells in different cell types and in samples. **(G)** Heatmap illustrating the chromatin accessibility and gene expression of 28,505 significantly (Pearson correlation $r > 0.45$ and adjusted p-value < 0.1, provided by chromVAR) linked peak-gene pairs.
