# Supplementary material for: Monkey multi-organ cell atlas exposed to estrogen
Source: Life Med. 2024 Mar 22;3(2):lnae012. doi: 10.1093/lifemedi/lnae012 (PMC11749546; doi:10.1093/lifemedi/lnae012)
Supplement: lnae012_suppl_Supplementary_Figs_S8 [file lnae012_suppl_Supplementary_Figs_S8.pdf]

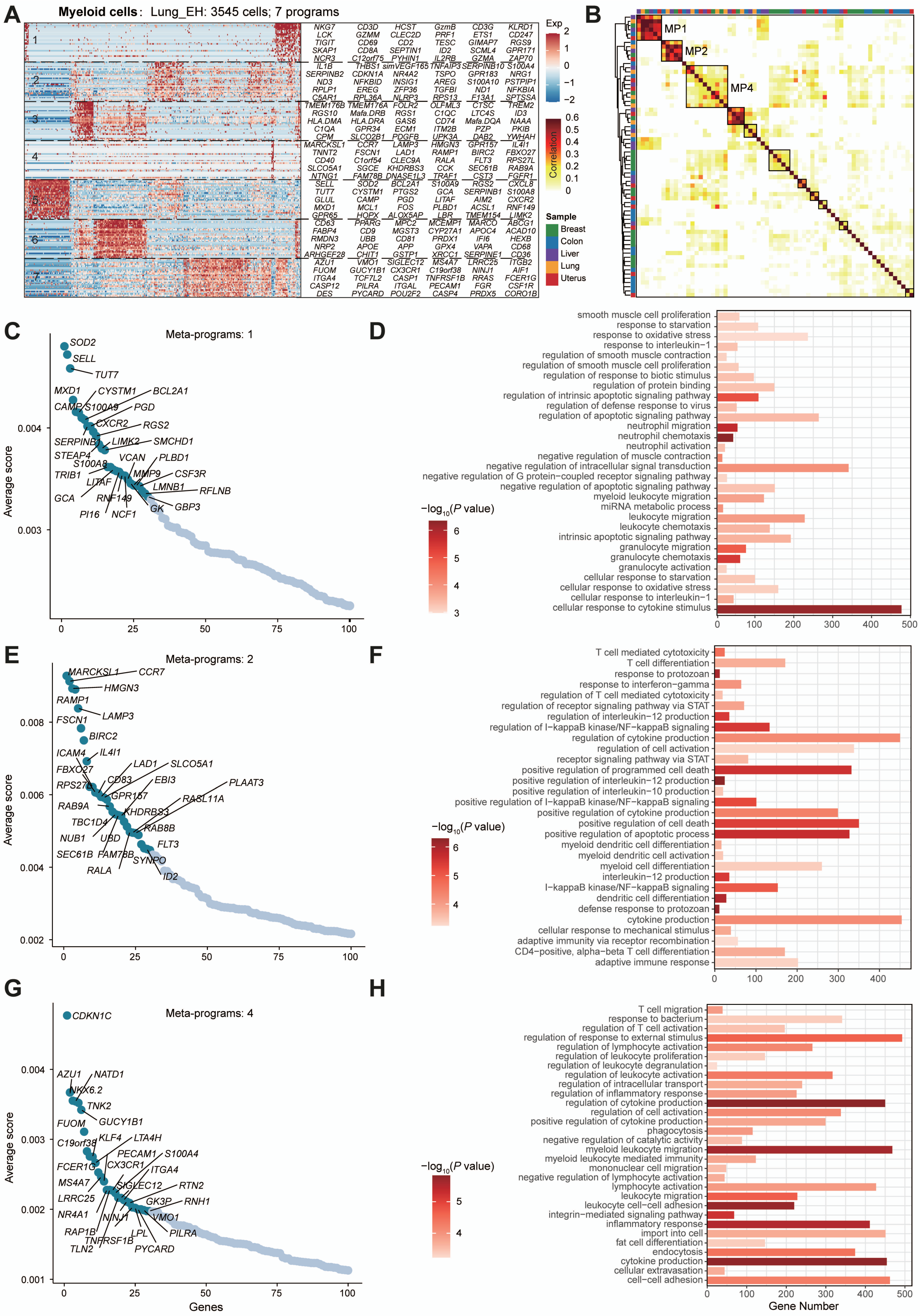

Supplementary Figure 8. Unbiased clustering reveals meta-programs that collectively respond to the estrogen signaling within each organ in myeloid cells. Related to Figure 2. (A) Heatmap showing gene expression programs deciphered from myeloid cells of a representative sample (Lung_EH) using NMF. **(B)** Heatmap showing pearson correlation indices for comparisons among 67 NMF programs based on their top 50 genes. Programs are ordered by clustering and grouped into MPs (marked by black solid lines). **(C)** Scatter plot showing the top 100 genes of MP1 ordered by average specific score and the top 30 genes with high scores are highlighted. **(D)** Bar plot displaying the functional pathways enriched by the top 100 genes in **(C)**. **(E)** Scatter plot showing the top 100 genes of MP2 ordered by average specific score and the top 30 genes with high scores are highlighted. **(F)** Bar plot displaying the functional pathways enriched by the top 100 genes in **(E)**. **(G)** Scatter plot showing the top 100 genes of MP4 ordered by average specific score and the top 30 genes with high scores are highlighted. **(H)** Bar plot displaying the functional pathways enriched by the top 100 genes in **(G)**.
