# Supplementary material for: Monkey multi-organ cell atlas exposed to estrogen
Source: Life Med. 2024 Mar 22;3(2):lnae012. doi: 10.1093/lifemedi/lnae012 (PMC11749546; doi:10.1093/lifemedi/lnae012)
Supplement: lnae012_suppl_Supplementary_Figs_S10 [file lnae012_suppl_Supplementary_Figs_S10.pdf]

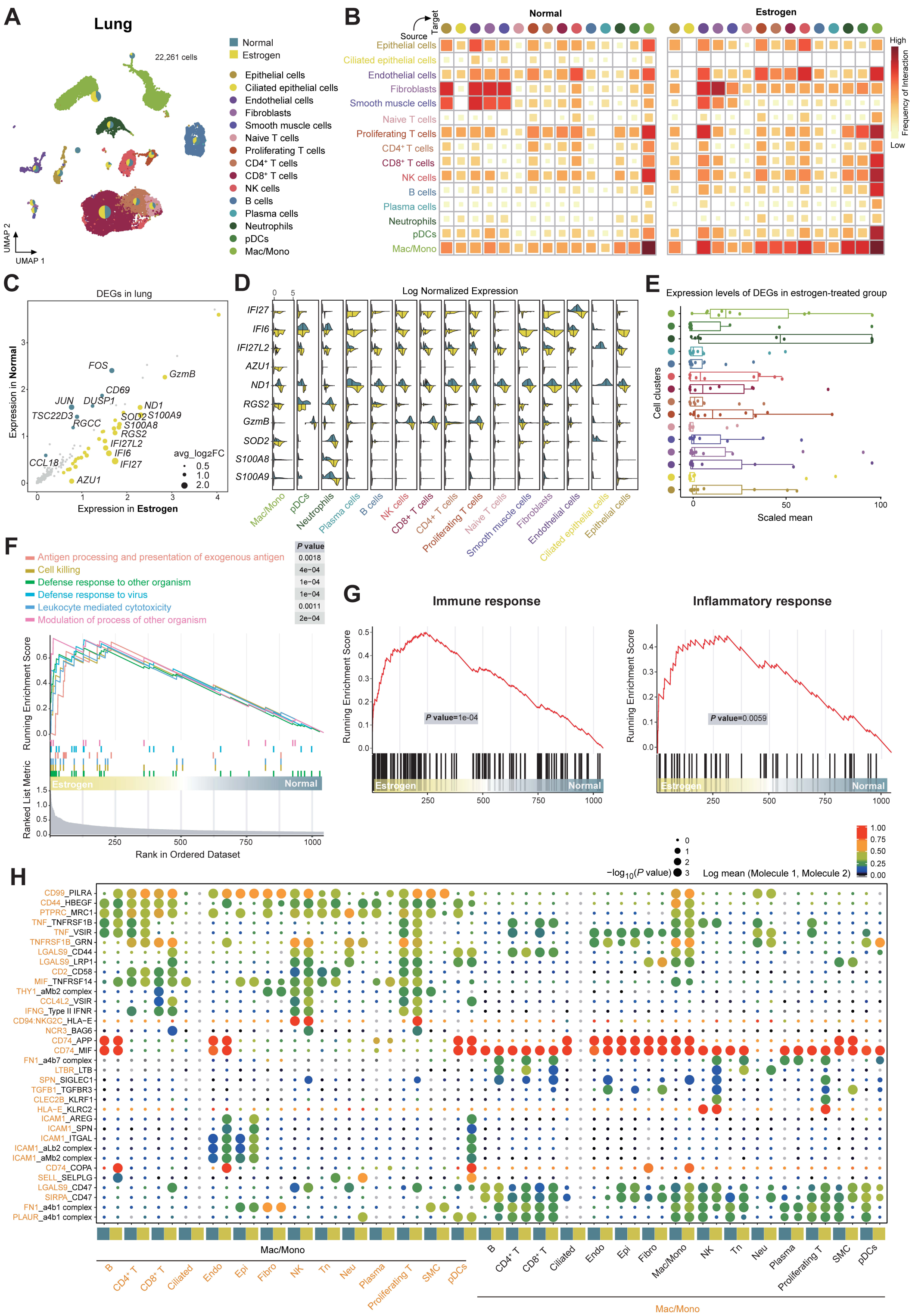

Supplementary Figure 10. Estrogen promotes the interaction between immune cells in lung tissue. (A) UMAP plot displaying the integrated cell map of lung, consisting of 22,261 cells from 15 annotated cell types. Cells are colored by cell types. Pie chart showing the proportion of two sample groups, estrogen-treated and normal groups, in each cell types. **(B)** Heatmap showing the cell-cell interaction intensity among lung cell types in two sample groups. Block sizes and colors are proportional to the interaction frequency. **(C)** Scatter plots showing DEGs between estrogen and normal group, and the top 10 DEGs are marked. Each dot represents a DEG, and its size is proportional to the fold change. (*P* value < 0.05, avg_$\log_2$FC > 0.5) **(D)** Stacked vlnplot showing the expression in each cell type and group. **(E)** Box plots show expression levels of DEGs in estrogen group. Cell clusters are all cell types annotated in lung. **(F)** Line chart showing significantly up-regulated top 6 GO terms of lung by GSEA. **(G)** GSEA of specific pathways related t o immunity a nd inflammation in lung. **(H)** Ligand-receptor interactions between Mac/Mono and all celltypes in lung. Each row represents a ligand-receptor pair, and each column defines a pair of cell-cell interaction. *P* values were calculated by CellPhoneDB without multiple comparisons.
