# Supplementary material for: Monkey multi-organ cell atlas exposed to estrogen
Source: Life Med. 2024 Mar 22;3(2):lnae012. doi: 10.1093/lifemedi/lnae012 (PMC11749546; doi:10.1093/lifemedi/lnae012)
Supplement: lnae012_suppl_Supplementary_Figs_S11 [file lnae012_suppl_Supplementary_Figs_S11.pdf]

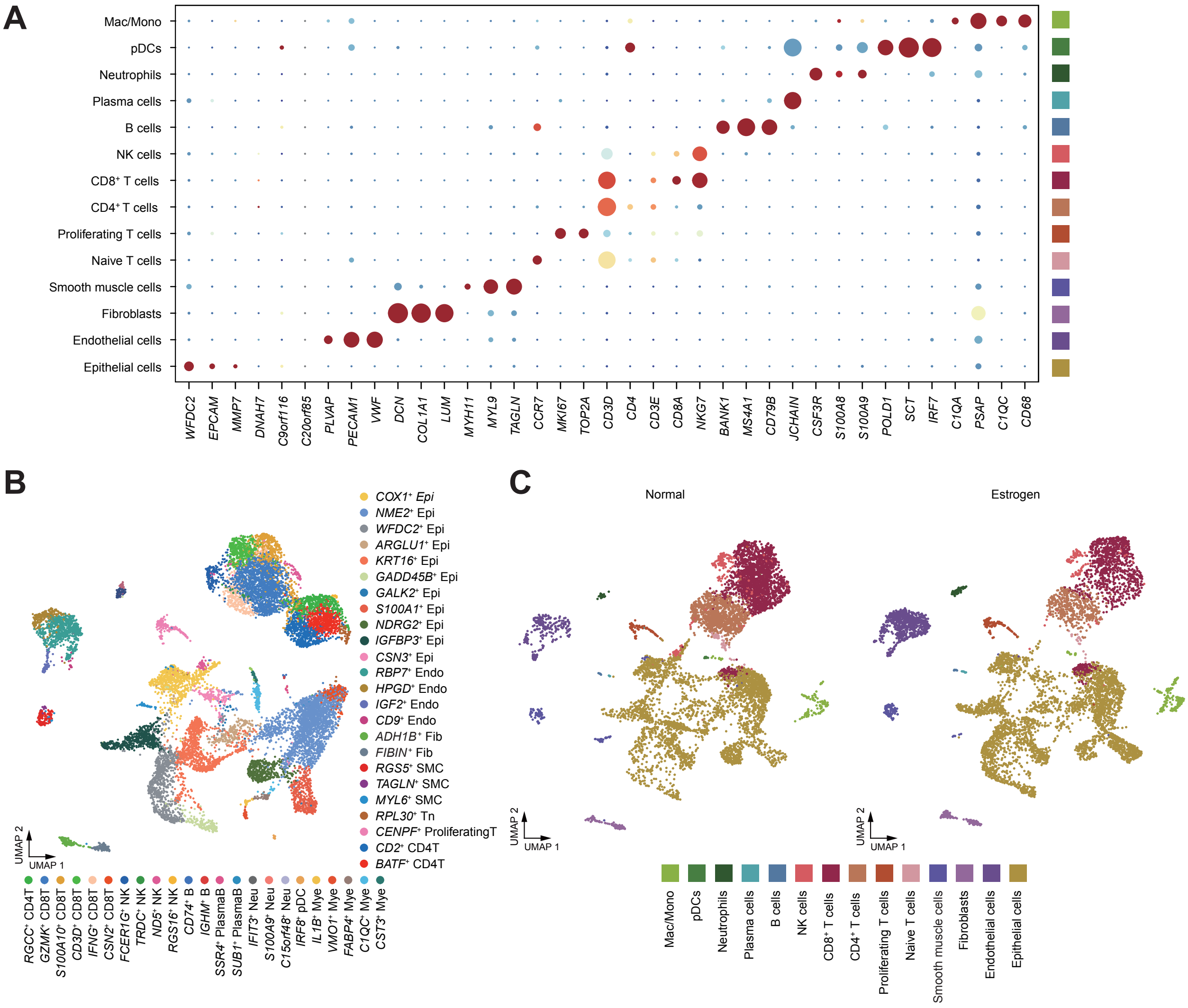


**Supplementary Figure 11. A supplement for scRNA-seq data analysis in breast. Related to Figure 5. (A)** Dot plot showing the representative marker genes of all the major cell types. **(B)** UMAP visualization of subclusters of the major cell types. **(C)** Cluster variation of major cell types comparing the normal and estrogen group.
