# Supplementary material for: Monkey multi-organ cell atlas exposed to estrogen
Source: Life Med. 2024 Mar 22;3(2):lnae012. doi: 10.1093/lifemedi/lnae012 (PMC11749546; doi:10.1093/lifemedi/lnae012)
Supplement: lnae012_suppl_Supplementary_Figs_S12 [file lnae012_suppl_Supplementary_Figs_S12.pdf]

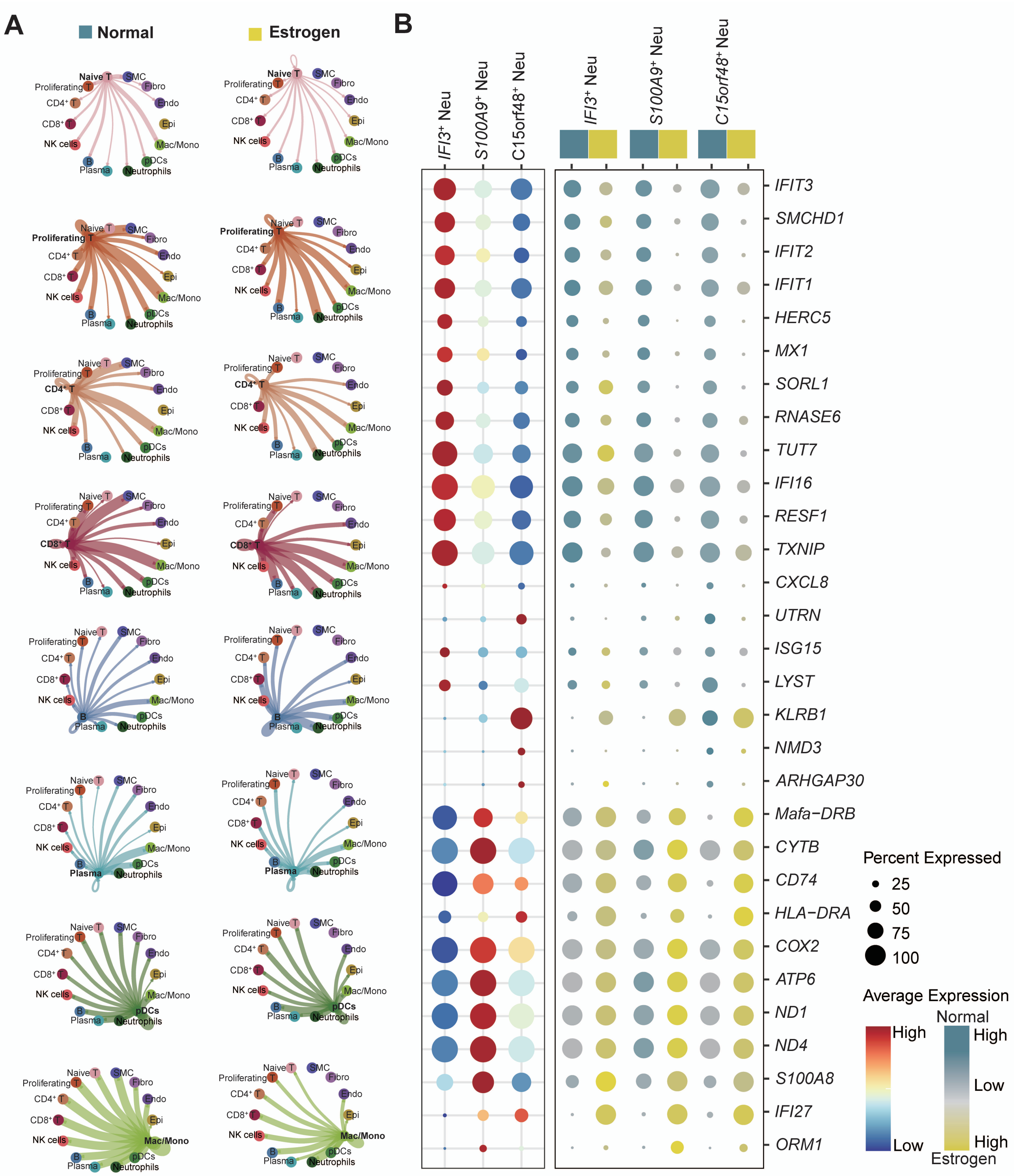

Supplementary Figure 12. Network of immune cells and neutrophil heterogeneity in the liver. Related to Figure 6. (A) Cell-cell interaction network in immune cells between two sample groups in the liver. The thickness represents the interaction frequency. **(B)** Dot plot showing expression levels of DEGs at the end of pseudotime **(Fig. 6J)** in respective neutrophil subtypes and groups.
