# Supplementary material for: Monkey multi-organ cell atlas exposed to estrogen
Source: Life Med. 2024 Mar 22;3(2):lnae012. doi: 10.1093/lifemedi/lnae012 (PMC11749546; doi:10.1093/lifemedi/lnae012)
Supplement: lnae012_suppl_Supplementary_Figs_S13 [file lnae012_suppl_Supplementary_Figs_S13.pdf]

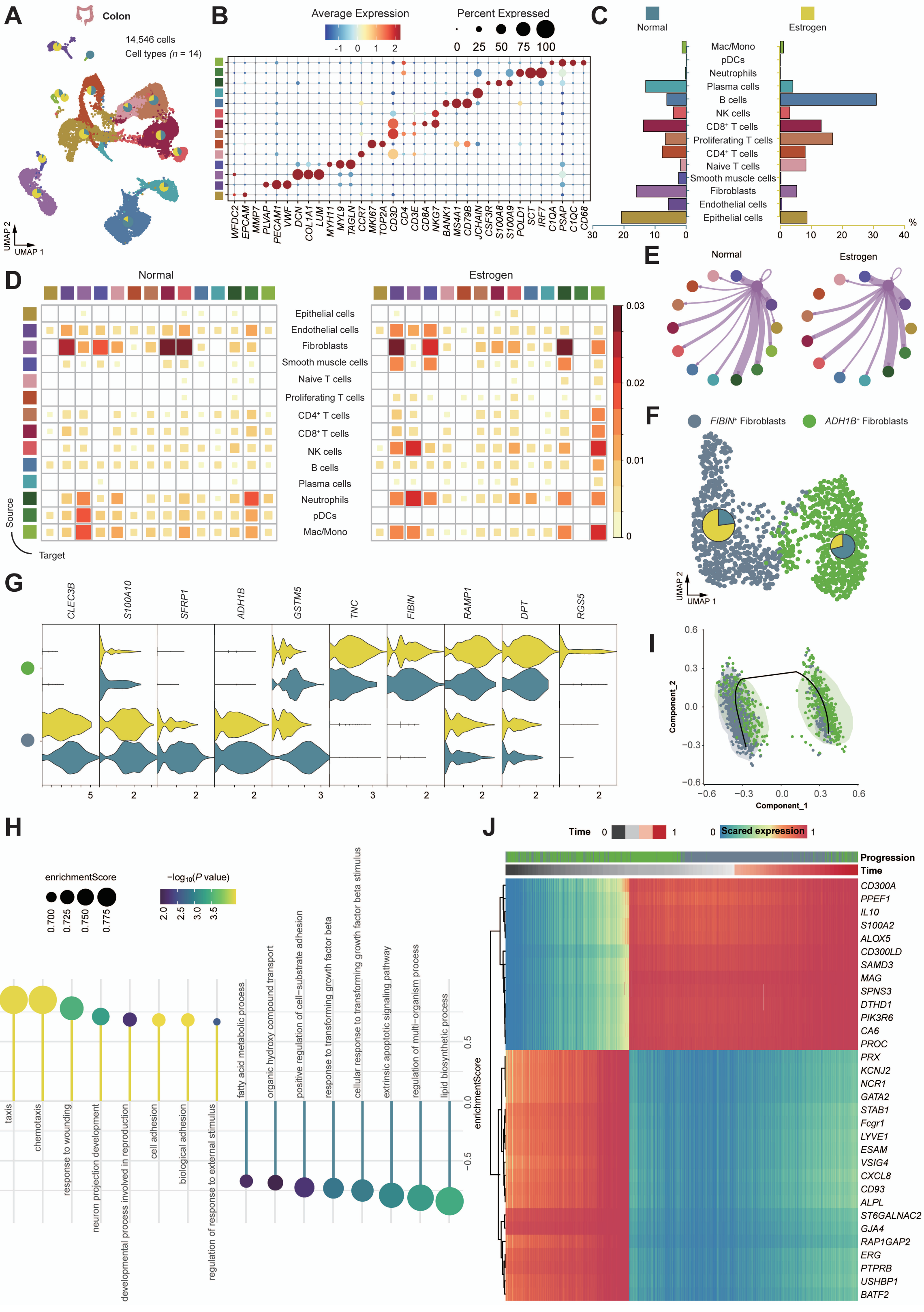

Supplementary Figure 13. Estrogen drives changes in fibroblast function and phenotype in colon tissue. (A) Distribution of 14 major cell subtypes on the UMAP. With pie chart showing the percentages in different groups. (B) Bar plot showing the proportion of Colon cell types. (C) Dot plots showing representative marker genes across the major cell types. (D) Heatmap showing the interaction network among major cell types constructed by CellPhoneDB. The width of the arrow represents the proportion of cells in a given cell type. (E) Ligand-receptor interactions between fibroblasts and other cell types in different groups. (F) Distribution of the fibroblast subtypes on the UMAP. (G) Stacked Vlnplot showing the expression of selected marker genes for the fibroblast subtypes. (H) The enrichment of functional pathways in fibroblast subclusters based on GO pathways enrichment analysis. (I) Pseudotime trajectory of fibroblast subclusters by SCORPIUS. An inferred trajectory was plotted. (J) The time series heatmap based on former Pseudotime trajectory analysis using SCORPIUS.
