# Supplementary material for: Monkey multi-organ cell atlas exposed to estrogen
Source: Life Med. 2024 Mar 22;3(2):lnae012. doi: 10.1093/lifemedi/lnae012 (PMC11749546; doi:10.1093/lifemedi/lnae012)
Supplement: lnae012_suppl_Supplementary_Figs_S14 [file lnae012_suppl_Supplementary_Figs_S14.pdf]

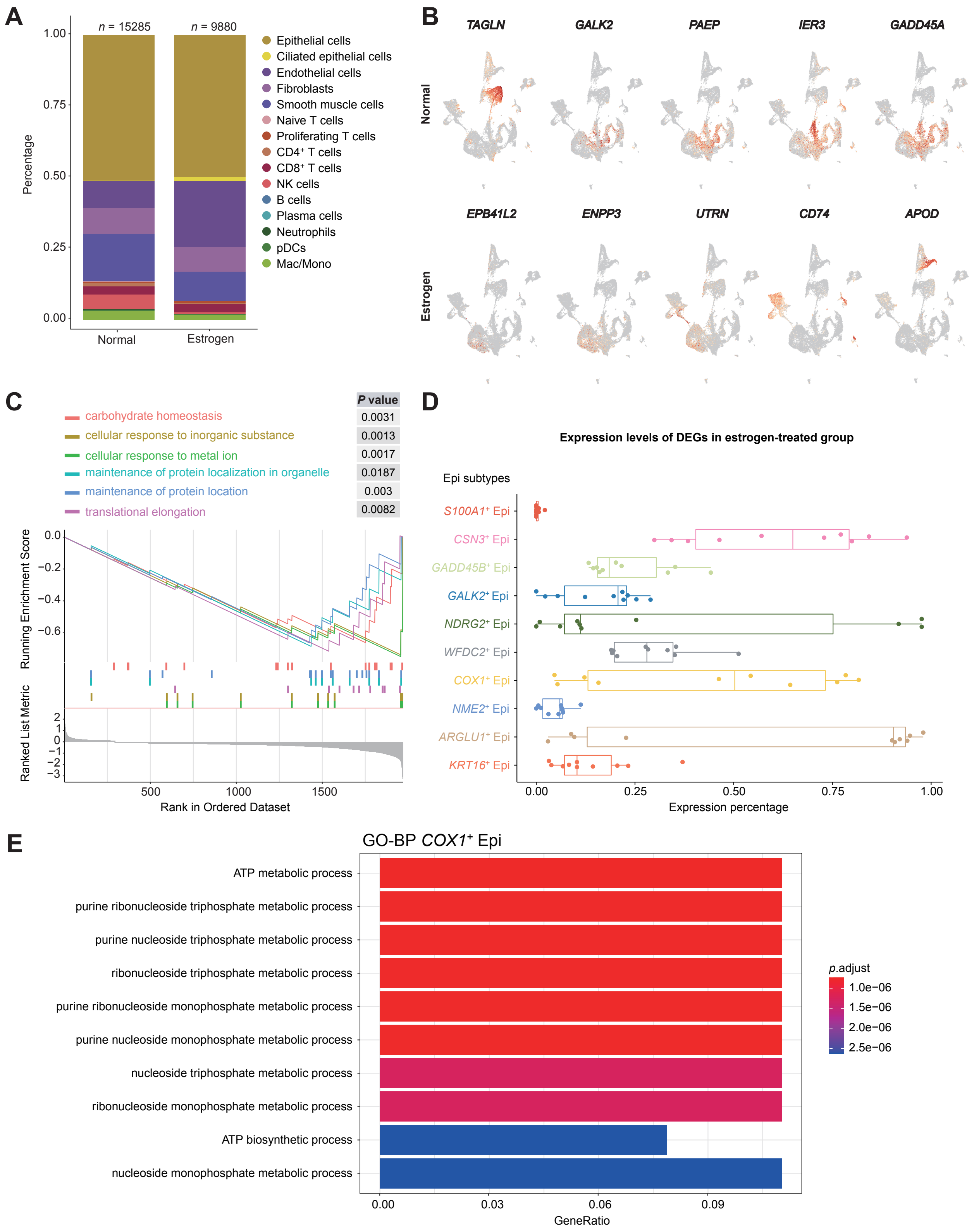

Supplementary Figure 14. Location and function of DEGs in uterus cell subtypes. Related to Figure 7. (A) Bar plot showing the percentage of cell types in the uterus, and "n" refers to the number of cells. **(B)** FeaturePlot showing the top 5-10 DEGs. **(C)** Line chart showing significantly down-regulated top 6 GO terms o f the uterus by GSEA. **(D)** Box plots show expression levels of DEGs in the estrogen group (*IFI6*, *CD109*, *LYZ*, *SRRM2*, *PNISR*, *EPB41L2*, *ENPP3*, *UTRN*, *CD74*, *APOD*). Epi subtypes are all subtypes annotated in the uterus epithelial cells. **(E)** Barplot showing the top 10 terms of BP enrichment terms for DEGs (*COX1*$^+$ epithelial cells *vs.* other epithelial cells) of epithelial cells in the uterus.
