# Supplementary material for: Monkey multi-organ cell atlas exposed to estrogen
Source: Life Med. 2024 Mar 22;3(2):lnae012. doi: 10.1093/lifemedi/lnae012 (PMC11749546; doi:10.1093/lifemedi/lnae012)
Supplement: lnae012_suppl_Supplementary_Figs_S9 [file lnae012_suppl_Supplementary_Figs_S9.pdf]

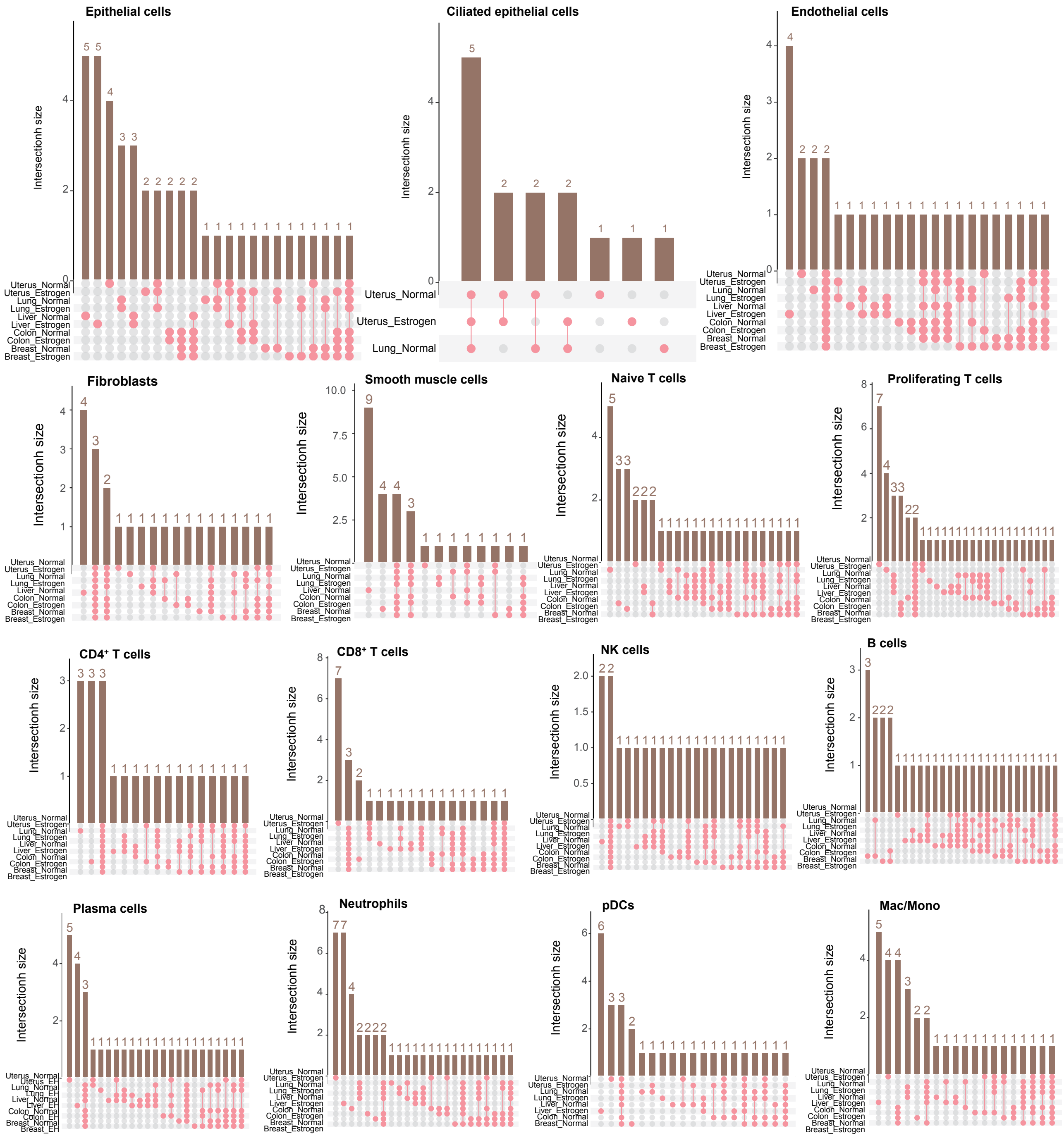

Supplementary Figure 9. Sample reproducibility of TF regulons across different cell types. Related to Figure 3. Upset plot showing the size of overlaps between the sets of organ-specific regulons identified in each cell type. The bar plot on the top shows the number of overlapping genes between two sets, or the number of unique genes in one set.
